# Effectiveness and safety of interleukin 6 receptor inhibitors versus conventional immunomodulatory therapy in steroid-refractory polymyalgia rheumatica

**DOI:** 10.1136/rmdopen-2026-006850

**Published:** 2026-08-03

**Authors:** Anisha Bharadwaj Dua, Robert F Spiera, Ara Dikranian, Cassandra Calabrese, Clifton O Bingham, Kerri Ford, Stefano Fiore, Lita Araujo, Natalia Petruski-Ivleva, Danielle L Isaman, Fenglong Xie, Jeffrey R Curtis

**Affiliations:** 1 Northwestern University Feinberg School of Medicine, Chicago, Illinois, USA; 2 Department of Medicine, Hospital for Special Surgery, New York City, New York, USA; 3 Cabrillo Center for Rheumatic Disease, San Diego, California, USA; 4 Department of Rheumatic and Immunologic Diseases, Cleveland Clinic, Cleveland, Ohio, USA; 5 Johns Hopkins University, Baltimore, Maryland, USA; 6 Sanofi, Cambridge, Massachusetts, USA; 7 Sanofi, Morristown, New Jersey, USA; 8 University of Alabama at Birmingham, Birmingham, Alabama, USA; 9 Foundation for Advancing Science, Technology, Education and Research, Birmingham, Alabama, USA

**Keywords:** Polymyalgia Rheumatica, Treatment, Methotrexate, Biological Therapy, Antirheumatic Agents

## Abstract

**Objective:**

To compare the effectiveness and safety of interleukin 6 receptor inhibitors (IL-6Ris) and conventional synthetic immunomodulators (csIM) in patients with polymyalgia rheumatica (PMR) who received glucocorticoids (GC) and initiated a new therapy for PMR.

**Methods:**

We evaluated the effectiveness and safety of IL-6Ris in a retrospective comparative cohort created from the US Medicare fee-for-service medical and part D prescription claims data. Patients with PMR, on GC, without prior IL-6Ri exposure and initiating IL-6Ri/csIM therapy were identified. These patients were categorised into csIM-naïve and csIM-experienced cohorts and matched using direct methods and propensity score methods. Primary endpoints were time-to-GC discontinuation and time-to-minimal GC use (≤2 mg/day or stop GC) through year 1. Incidence rates (IRs) for adverse events of special interest were reported for IL-6Ri and csIM initiators through year 2.

**Results:**

We included 415 matched treatment pairs (187/228, csIM-naïve/csIM-experienced). IL-6Ri initiators were more likely to discontinue GC (adjusted HR (aHR) 1.28, 95% CI 1.02 to 1.60; p=0.031) or achieve minimal GC use (aHR 1.28, 95% CI 1.03 to 1.58; p=0.025) by year 1 versus csIM initiators. At year 1, IR (95% CI)/100 patient-years for primary hospitalised infections was higher for IL-6Ri initiators versus csIM initiators (12.2 (95% CI 8.9 to 16.3) vs 5.7 (95% CI 3.6 to 8.6)); however, it was similar for year 2 (5.8 (95% CI 3.3 to 9.6) vs 6.4 (95% CI 3.8 to 10.1)). For other events of interest, the IRs were low and comparable in both groups.

**Conclusions:**

IL-6Ri therapy was more effective as a steroid-sparing agent than csIM for PMR with no new safety findings.

**WHAT IS ALREADY KNOWN ON THIS TOPIC**

- Many patients with polymyalgia rheumatica (PMR) experience relapses on or off glucocorticoids (GCs) or cannot tolerate GCs.
- Conventional synthetic immunomodulatory (csIM) therapies are frequently used for these patients as GC-sparing regimens, most commonly methotrexate (MTX), with limited evidence of their efficacy.
- Interleukin 6 receptor inhibitor (IL-6Ri) therapy has exhibited efficacy for PMR in multiple randomised controlled trials, and sarilumab, an IL-6Ri, has been approved for PMR.

**WHAT THIS STUDY ADDS**

- This is the largest real world study evaluating the effectiveness and long-term safety profile of IL-6Ri therapy in patients with PMR and the first study to compare IL-6Ri with csIM.
- Patients initiating IL-6Ri therapy were more likely to discontinue GCs and achieve minimal GC use (≤2 mg/day or stop GC) by year 1 versus any csIM or MTX alone.

**HOW THIS STUDY MIGHT AFFECT RESEARCH, PRACTICE OR POLICY**

- Based on these results, IL-6Ri therapy should be considered for patients with PMR who experience relapse or cannot tolerate GCs.

## Introduction

 Polymyalgia rheumatica (PMR) is a common inflammatory rheumatic condition that primarily affects older people, particularly those aged ≥50 years, with incidence increasing with age.^1 2^ It is characterised by elevated levels of acute-phase reactants, with neck, bilateral shoulder and hip pain, along with morning stiffness,^2 3^ which significantly impacts patients’ quality of life and physical function.^4 5^ Glucocorticoids (GCs) remain the current standard of care for PMR;^6 7^ however, relapses and extended GC use are frequently observed.^8–10^ A meta-analysis of 21 studies revealed that 43% patients with PMR experienced ≥1 relapse within 1 year of treatment initiation, and 77%, 51% and 25% patients remained on GCs at years 1, 2 and 5 after diagnosis, respectively.^11^ GC use is also associated with toxicity risk, particularly in elderly patients who may not be able to tolerate GC due to the high prevalence of age-related comorbidities, which in turn may be exacerbated by GC use.^12 13^ Prolonged GC exposure has been associated with an increased risk of serious adverse effects, such as infections, fractures, diabetes, heart failure and all-cause mortality.^8 14^ For GC-dependent patients, conventional synthetic immunomodulator (csIM) therapies are often used, although the data supporting their use are limited. Varying efficacy of methotrexate (MTX) as a steroid-sparing agent has been reported, resulting in its conditional use for PMR management (2015 Recommendations for the management of PMR: a European League Against Rheumatism/American College of Rheumatology collaborative initiative).^7 15–18^

Treatments targeting the interleukin-6 (IL-6) pathway have recently emerged as an option for patients with PMR who are GC-dependent or GC-intolerant. IL-6 is a key driver of the systemic inflammatory response and is implicated in the pathogenesis of PMR.^19^ Several small observational and randomised prospective studies on tocilizumab and sarilumab, both IL-6 receptor inhibitors (IL-6Ris), have demonstrated their significant GC-sparing effect, ability to achieve GC-free remission and reduced relapse rates among patients with PMR.^19–22^ The placebo-controlled sarilumab study—SAPHYR formed the basis for the US Food and Drug Administration (FDA) approval of sarilumab for the treatment of adult patients with PMR for whom GCs have proved inadequate or who cannot tolerate GC taper.^23^ However, the premature termination of the study because of recruitment challenges due to the COVID-19 pandemic limited the sample size.^22^ Furthermore, the comparator arm in the study comprised GC taper without csIM therapy.

To address these limitations and provide evidence supporting the effectiveness of sarilumab for supplemental biologics licence application to the US FDA, we conducted a real world evidence study among patients with PMR who were identified from the Medicare Chronic Conditions Warehouse and received GCs followed by treatment initiation with an IL-6Ri or csIM therapy. The study compared the effectiveness and safety of these therapies when added to GCs for csIM-naïve and csIM-experienced patients with PMR.

## Methods

### Study design

**Figure 1:**
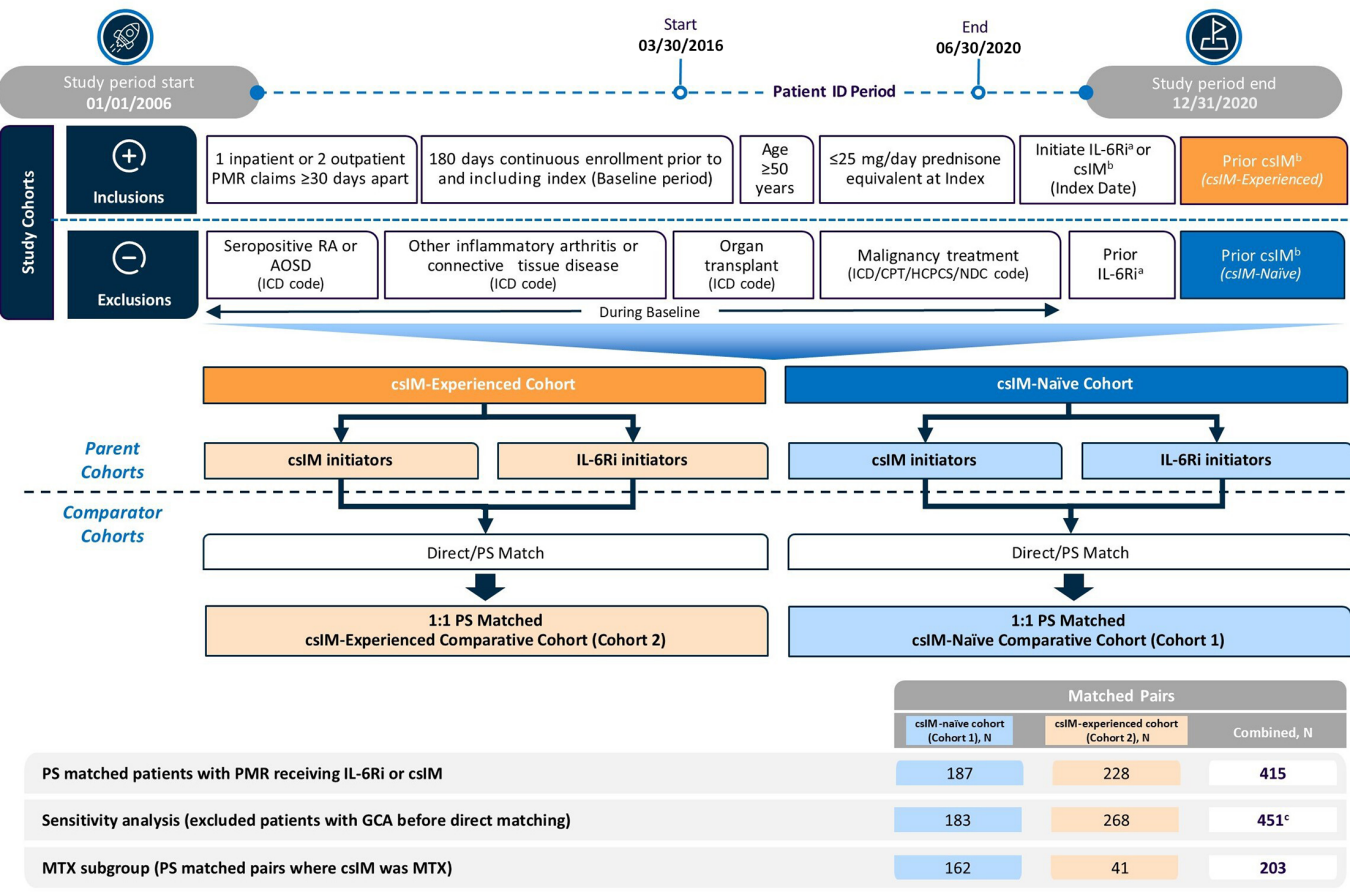
Study design ^a^sarilumab, tocilizumab; ^b^methotrexate, leflunomide, azathioprine ^c^by excluding patients with GCA before direct matching, the number of direct match criteria decreased, resulting in higher number of direct matches for the sensitivity analysis. AOSD, adult-onset still’s disease; csIM, conventional synthetic immunomodulators; CPT, current procedural terminology; GCA, giant cell arteritis; HCPCS, healthcare common procedure coding system; ICD-10, International Classification of Diseases, Tenth Revision; ID, identification; IL-6Ri, IL-6 receptor inhibitors; MTX, methotrexate; NDC, national drug code; PMR, polymyalgia rheumatica; PS, propensity score; RA, rheumatoid arthritis.

This study employed an observational, retrospective, comparative cohort design and used the US Medicare fee-for-service medical and part D prescription claims data from 1 January 2006 to 31 December 2020. The US Medicare data provide a comprehensive, single-payer view; cover >94% of individuals in the USA aged ≥65 years and younger patients who are disabled or who have certain end-stage diseases; and include data on demographics, diagnoses and codes for procedures performed during outpatient visits or inpatient stays, and outpatient prescription fill records. Patients on GC who initiated IL-6Ri (tocilizumab or sarilumab) or csIM (MTX, leflunomide (LEF), azathioprine) therapy between 30 March 2016 and 30 June 2020 were identified. IL-6Ri and csIM therapies were presumed to be added because patients were either GC-dependent or needed to reduce GC exposure due to intolerability. Patients without prior csIM exposure were included in the csIM-naïve cohort (cohort 1), whereas those with prior csIM exposure were included in the csIM-experienced cohort (cohort 2). For the csIM comparator group, the index csIM was required to be different from the prior csIM. The date of therapy initiation was designated as the index date. In each cohort, the outcomes were compared between IL-6Ri initiators and csIM initiators (figure 1 and online supplemental table 1). For effectiveness, a subgroup analysis comparing results of IL-6Ri therapy versus MTX, rather than all csIMs, was also conducted. The follow-up periods to evaluate effectiveness and safety were 1 year and 2 years, respectively.

### Study population

Patients aged >50 years with at least one inpatient or two outpatient claims for PMR (International Classification of Diseases (ICD)−9-clinical modification (CM)=725; ICD-10-CM=M35.3) that were ≥30 days apart were included. Patients with giant cell arteritis (GCA) often develop PMR; thus, patients with a history of GCA were not excluded. Patients were required to have a minimum baseline period of 180-day continuous enrolment prior to and including index day and ≥1 day of follow-up after index. Oral GC prednisone-equivalent dose (PED) of ≤25 mg/day at index was required to exclude those potentially being treated for GCA with higher doses of GC. Patients who had received prior IL-6Ri therapy were excluded. Exclusions also applied to the following patients: those initiating IL-6Ri and csIM therapies on the same day, and those with prior claims during the baseline period for seropositive rheumatoid arthritis (RA), adult-onset Still’s disease, other inflammatory arthritis or connective tissue disease, organ transplant, multiple sclerosis, or active treatment for malignancy. Definitions for baseline exclusion measures and each variable are presented in online supplemental tables 2 and 3, respectively.

In each cohort, csIM initiators were direct-matched to IL-6Ri initiators (up to 3:1) based on age (±3 years), sex, any history of GCA, baseline period daily GC dose (seven categories from <2.5 mg to >25.0 mg PED), daily GC dose at index (six categories: <2.5 mg to 20.0–25.0 mg PED) and temporal proximity of prior csIM therapy (time from prior csIM use to index; 1–60, 61–180, >180 days (in cohort 2 only)). History of GCA and baseline daily GC dose were added as the direct-matching criteria, and the number of baseline and index daily GC dose categories was increased (previously 3: <5, 5–10, >10 mg PED), following a protocol amendment due to imbalances observed using these original categories.

Following direct-matching without replacement, 1:1 propensity score (PS) matching without replacement was performed to control for baseline confounding. Additional PS-matching covariates included race, region, calendar year of index treatment, reason for entering Medicare, Charlson Comorbidity Index (CCI) category, time from first PMR diagnosis to index, baseline healthcare resource utilisation (number of inpatient days, emergency department visits, outpatient visits), conditions that may be associated with GC use (asthma, atopic dermatitis, chronic obstructive pulmonary disease, Crohn’s disease, psoriasis, ulcerative colitis) and seronegative RA (definition of each variable is provided in online supplemental table 3). For the subgroup analysis of IL-6Ri therapy and MTX, PS-matched pairs from the MTX subgroup (csIM therapy) were retained for comparison.

Although the patients were matched based on their history of GCA, it was assumed that inclusion may confound the results or limit their generalisability. Therefore, a sensitivity analysis was conducted which excluded patients with history of GCA before direct matching.

### Effectiveness outcomes

The two effectiveness primary outcomes were as follows: (1) Complete discontinuation of oral GC therapy, and (2) Composite outcome of minimal GC use (≤2 mg PED/day or stop GC). A threshold of 2 mg PED/day was selected based on the limited available data, suggesting low risk of serious GC-related adverse events at this dose.^24^ Both outcomes were assessed for 1 year and evaluated using Kaplan-Meier curves and HRs estimated using Cox proportional hazards models.

Secondary outcomes included cumulative GC dose over 52 weeks and persistence of index PMR therapy (cohort 1 only) assessed up to 1 year. Cumulative GC dose was defined as the average weekly GC dose received over the available follow-up period through a maximum of 1 year, calculated as total concentration (mg) of PED dispensed over follow-up/days of follow-up observation multiplied by 7. Follow-up was defined as 1 day after index until the earliest of the following events: 60 days prior to loss of Medicare coverage, stopping index medication (>60 day gap), switching or adding another PMR treatment, death, at the end of evaluation period or 1 year (day 365).

Persistence was defined as not having a treatment gap of >60 days; for the csIM group, not switching to/adding a new csIM or IL-6Ri; and for the IL-6Ri group, not switching to/adding a csIM after the index date. Persistence was evaluated only for cohort 1 because in cohort 2, the patients were expected to continue therapy or add additional treatment(s) for PMR due to limited alternatives. The evaluation of persistence was limited to 1 year to align with the Cox proportional hazards assumption and with the recommended duration of treatment for steroid-sparing therapy.^25 26^

### Safety outcomes

Primary outcomes included adverse events of special interest (AESIs) comprising hospitalised infections (primary diagnosis), gastrointestinal (GI) perforation, major adverse cardiovascular events, malignancy and drug-induced liver injury (DILI). They were assessed as incidence rates (IRs). Secondary outcomes included combined AESIs, hospitalised infections (any diagnosis position), any infection (hospitalised (any diagnosis position) or outpatient) and all-cause hospitalisations, and they were assessed as HRs comparing IL-6Ri initiators with csIM initiators (variable definition provided in online supplemental table 3). Primary and secondary endpoints were assessed from index to year 1 (days 1–365), index to year 2 (days 1–730), and year 1 to year 2 (days 366–730, with and without reindexing patients at day 366). Except for hospitalised infections, patients with prior events of the same type were excluded from each outcome-specific analysis due to challenges in distinguishing between a recurrent event and follow-up for the prior event of that type. Secondary infections occurring within 7 days of discharge were considered as a single event.

For the year 1 to year 2 analysis without reindexing, the patients who experienced an event in the first year (days 1–365) were excluded from the at-risk pool. With reindexing, these patients were reindexed on day 366 and included in the at-risk pool for analysis.

Coding algorithms developed using the ICD (ICD-9/10), the healthcare common procedure coding system, the current procedural terminology and the national drug code for safety events followed established conventions and were informed by multiple previous studies demonstrating high validity versus standard medical record reviews.^27^

### Statistical analysis

Baseline characteristics of PS-matched IL-6Ri and csIM initiators in cohorts 1 and 2 as well as study endpoints during follow-up were summarised using counts with percentages for dichotomous and categorical measures, and means with SD and medians with IQRs for continuous measures. Covariate balance for PS-matched cohorts was assessed by evaluating standardised mean differences (SMDs) between groups for each matching factor. Covariates with an SMD of >0.10 were considered imbalanced.^28^ For considering the matched cohorts exchangeable, 90% covariates included in the PS model were required to be balanced across the PS-matched study groups.

The effectiveness primary endpoints were assessed using a Cox proportional hazards model to compare the time-to-GC discontinuation and time-to-minimal GC use in cohorts 1 and 2. The Cox models estimated HRs with 95% CIs and were reported as a combined effect estimate stratified by csIM exposure (naïve (cohort 1) versus experienced (cohort 2)). In addition to achieving the outcome, the patients were censored if they lost Medicare coverage (enrolment end − 60 days), stopped their index medication (>60 days gap), switched to or added another PMR treatment, died or at the end of the evaluation period, that is, after 1 year (day 365). Outcome models were adjusted for any residual imbalances in either cohort after PS matching for any covariates assessed in both cohorts with SMDs of >0.10.

Pearson’s χ^2^ test, Wilcoxon rank-sum test or Fisher’s exact test were also used because the sample size for the subgroup analysis of IL-6Ri and MTX therapies was relatively small, and SMDs may over-represent covariate imbalance when the data are sparse. In this analysis, the covariates were considered imbalanced and in need of further outcome model adjustment only if SMD was >0.10 and the value of p was <0.05.

The secondary effectiveness endpoint, that is, average weekly GC dose up to 1 year, was assessed using Wilcoxon’s rank-sum test. The patients were censored for the reasons described above, except for the effectiveness primary outcomes. Statistical significance for all comparative analyses was set at α=0.05. To better understand the differences in GC use over time, an analysis was conducted that evaluated the difference in mean change in weekly PED for IL-6Ri initiators versus csIM initiators over 3-month intervals. Weighted linear regression was used to evaluate the potential for a difference in GC dose reduction in the IL-6 initiators relative to the csIM initiators in 90-day intervals, calculating weights based on the inverse of the width of the 95% CI, as well as assessing for a statistical trend across the four 90-day intervals.

IRs for safety events were reported per 100 patient-years (PYs). For primary safety endpoints with <5 events, the exact Poisson method was used to calculate the 95% CIs. A Cox proportional hazards model was used to estimate HRs for secondary safety endpoints. The Cox models were initially intended to be adjusted for infections only due to the limited number of other events. However, to maintain consistency in comparing event rates, adjustments for infections were not made.

SAS V.9.4 was used for all analyses. The results from cohorts 1 and 2 were pooled using the STRATA option in the SAS PHREG procedure for both effectiveness and safety outcomes. To protect patient privacy, any cell with a non-zero result <11 was shown as <11, and if any cell could be used to back calculate or otherwise derive the result, it was shown as ‘redacted’ to prevent any back calculation.

## Results

### Selection of PMR cohort and IL-6Ri initiators

A total of 663 IL-6Ri initiators met the inclusion criteria: 251 for cohort 1 and 412 for cohort 2. Of these, 187 and 228 IL-6Ri initiators from cohorts 1 and 2, respectively, were direct matched to 502 and 463 csIM initiators, respectively. All direct-matched IL-6Ri initiators were included in the analysis resulting in 187 PS-matched pairs in cohort 1 and 228 PS-matched pairs in cohort 2. For the subgroup analysis of IL-6Ri versus MTX therapies, 162 PS-matched pairs were included in cohort 1 and 41 pairs in cohort 2 (online supplemental table 1).

### Patient characteristics

Patient characteristics for the csIM group (both cohorts 1 and 2) were similar prior to PS matching versus post PS matching, with a few exceptions. Presence of GCA (7.0% vs 13.9%) and the time from first PMR diagnosis code to index (247 vs 348 days) were lower prior to PS matching than post PS matching in cohort 1; in cohort 2, the presence of seronegative RA during the baseline period (54.4% vs 69.7%) was lower prior to PS matching (table 1 and online supplemental table 4). In both PS-matched cohorts 1 and 2, the mean age of the patients was approximately 75 years, >70% were female and >85% white. The most common index csIM for patients in cohorts 1 and 2 was MTX (86.6%) and LEF (71.1%), respectively. MTX was the most common prior csIM in cohort 2 of both IL-6Ri and csIM groups (89.9% and 79.4%, respectively) (table 1).

**Table 1:** Characteristics of patients with PMR receiving IL-6Ri or csIM therapy in the csIM-naïve or csIM-experienced cohorts after PS-matching

Characteristics	csIM-naïve cohort (cohort 1)			csIM-experienced cohort (cohort 2)		
	IL-6Ri (n=187)	csIM (n=187)	SMD	IL-6Ri (n=228)	csIM (n=228)	P value
Treatment						
Index csIM, n (%)						
Methotrexate	0 (0.0%)	162 (86.6%)		0 (0.0%)	41 (18.0%)	
Leflunomide	0 (0.0%)	Redacted		0 (0.0%)	162 (71.1%)	
Azathioprine	0 (0.0%)	<11		0 (0.0%)	25 (11.0%)	
Prior csIMs,* n (%)						
No. of prior csIMs, mean (SD)	–	–		1.2 (0.4)	1.0 (0.1)	p<0.001
Methotrexate	–	–		205 (89.9%)	181 (79.4%)	p=0.002
Leflunomide	–	–		55 (24.1%)	Redacted	p=0.171
Azathioprine	–	–		12 (5.3%)	<11	p=0.503
PS-matched covariates			SMD			SMD
Temporal proximity of prior csIM use, days, n (%)						0.12
1–60	–	–		92 (40.4%)	90 (39.5%)	
61–180	–	–		51 (22.4%)	42 (18.4%)	
>180	–	–		85 (37.3%)	96 (42.1%)	
Age at index date, years, mean (SD)	75.7 (6.8)	76.1 (6.1)	0.06	74.0 (5.9)	74.5 (5.5)	0.09
Sex, female, n (%)	136 (72.7%)	136 (72.7%)	0.00	162 (71.1%)	174 (76.3%)	0.12
Baseline daily GC dose category (mg), n (%)			0.16			0.06
<2.5	13 (7.0%)	16 (8.6%)		24 (10.5%)	26 (11.4%)	
2.5–<5.0	24 (12.8%)	25 (13.4%)		54 (23.7%)	54 (23.7%)	
5–<10	65 (34.8%)	72 (38.5%)		109 (47.8%)	111 (48.7%)	
10–<15	51 (27.3%)	44 (23.5%)		30 (13.2%)	26 (11.4%)	
15–<20	19 (10.2%)	14 (7.5%)		<11	<11	
20–25	<11	<11		<11	<11	
>25	<11	Redacted		0	0	
Index daily GC dose category (mg), n (%)			0.10			0.12
<2.5	<11	<11		<11	<11	
2.5–<5.0	Redacted	Redacted		Redacted	Redacted	
5–<10	45 (24.1%)	53 (28.3%)		80 (35.1%)	87 (38.2%)	
10–<15	44 (23.5%)	40 (21.4%)		59 (25.9%)	57 (25.0%)	
15–<20	43 (23.0%)	41 (21.9%)		31 (13.6%)	30 (13.2%)	
20–25	33 (17.6%)	33 (17.6%)		31 (13.6%)	34 (14.9%)	
GCA, n (%)	26 (13.9%)	26 (13.9%)	0.00	<11	<11	0.00
Race, n (%)			0.12			0.04
White	161 (86.1%)	163 (87.2%)		212 (93.0%)	214 (93.9%)	
Black	<11	12 (6.4%)		<11	<11	
Other	Redacted	12 (6.4%)		<11	<11	
Region, n (%)			0.09			0.11
West	45 (24.1%)	43 (23.0%)		47 (20.6%)	54 (23.7%)	
Midwest	Redacted	Redacted		53 (23.2%)	49 (21.5%)	
North-east	60 (32.1%)	65 (34.8%)		56 (24.6%)	48 (21.1%)	
South	62 (33.2%)	56 (29.9%)		72 (31.6%)	77 (33.8%)	
Unknown	<11	<11		0 (0.0%)	0 (0.0%)	
Original reason for Medicare eligibility, n (%)			0.05			0.02
Age ≥65 years	166 (88.8%)	169 (90.4%)		194 (85.1%)	192 (84.2%)	
Disabled	21 (11.2%)	18 (9.6%)		34 (14.9%)	36 (15.8%)	
Charlson Comorbidity Index **category, n (%)**			0.13			0.08
0–1	82 (43.9%)	71 (38.0%)		97 (42.5%)	88 (38.6%)	
2–3	68 (36.4%)	79 (42.2%)		87 (38.2%)	91 (39.9%)	
≥4	37 (19.8%)	37 (19.8%)		44 (19.3%)	49 (21.5%)	
Time from first PMR diagnosis to index, median days (IQR)	295.0 (133.0 to 717.5)	358.0 (147.0 to 811.0)	0.04	704.0 (278.8 to 1438.5)	644.5 (294.8 to 1333.3)	0.01
Baseline inflammatory conditions, n (%)						
Asthma	16 (8.6%)	21 (11.2%)	0.09	26 (11.4%)	22 (9.6%)	0.06
Atopic dermatitis	<11	<11	0.06	<11	<11	0.00
COPD	16 (8.6%)	20 (10.7%)	0.07	17 (7.5%)	17 (7.5%)	0.00
Crohn’s disease	0	0	0.00	<11	<11	0.11
Psoriasis	<11	0 (0.0%)	0.10^†^	<11	<11	0.00
UC	0	0	0.00	<11	<11	0.05
Seronegative RA	59 (31.6%)	55 (29.4%)	0.05	160 (70.2%)	159 (69.7%)	0.01
Baseline number of inpatient days			0.09			0.03
Mean (SD)	1.1 (4.4)	0.8 (2.5)		0.7 (2.9)	0.7 (2.2)	
Median (IQR)	0.0 (0.0 to 0.0)	0.0 (0.0 to 0.0)		0.0 (0.0 to 0.0)	0.0 (0.0 to 0.0)	
Baseline number of emergency department visits			0.07			0.09
Mean (SD)	0.4 (1.1)	0.5 (1.1)		0.4 (0.9)	0.5 (1.1)	
Median (IQR)	0.0 (0.0 to 0.5)	0.0 (0.0 to 1.0)		0.0 (0.0 to 0.0)	0.0 (0.0 to 0.0)	
Baseline number of outpatient office visits			0.01			0.00
Mean (SD)	10.3 (5.7)	10.3 (5.3)		9.9 (5.3)	9.9 (5.5)	
Median (IQR)	9.0 (6.0, 13.0)	9.0 (6.0, 14.0)		9.0 (6.0, 12.0)	9.0 (6.0, 13.3)	
Calendar year of index date, n (%)			0.12			0.08
2016	<11	12 (6.4%)		27 (11.8%)	23 (10.1%)	
2017	41 (21.9%)	38 (20.3%)		71 (31.1%)	70 (30.7%)	
2018	55 (29.4%)	63 (33.7%)		65 (28.5%)	63 (27.6%)	
2019	56 (29.9%)	54 (28.9%)		50 (21.9%)	57 (25.0%)	
2020	Redacted	20 (10.7%)		15 (6.6%)	15 (6.6%)	

* No SMD, value of p analysed by the Wilcoxon rank-sum test; Pearson’s χ2 test.

† SMD >0.10 rounded to 0.10.

COPD, chronic obstructive pulmonary disease; csIM, conventional synthetic immunomodulator; GC, glucocorticoid; GCA, giant cell arteritis; IL-6Ri, interleukin 6 receptor inhibitor; PMR, polymyalgia rheumatica; PS, propensity score; RA, rheumatoid arthritis; SMD, standardised mean difference; UC, ulcerative colitis.

In the subgroup analysis evaluating MTX versus IL-6Ri initiators, the most common prior csIM for cohort 2 was MTX (92.7%, 38/41) for IL-6Ri initiators and LEF (90.2%, 37/41) for MTX initiators (online supplemental table 5).

For both cohorts, most covariates (13/22) were comparable after PS matching; however, based on an SMD of >0.10, few relatively small residual imbalances were observed between the groups. Of these, sex, race, region, baseline and index day PED category, calendar year of index date, Crohn’s disease, psoriasis and CCI category had an SMD of >0.10 in either one or both cohorts (table 1) and were included in the combined Cox proportional hazards outcomes model.

The subgroup analysis of IL-6Ri versus MTX groups revealed that the SMD for some covariates was >0.10; however, none of these covariates had a value of p<0.05, so they were not deemed as imbalanced (online supplemental table 5).

### Effectiveness outcomes

**Figure 2:**
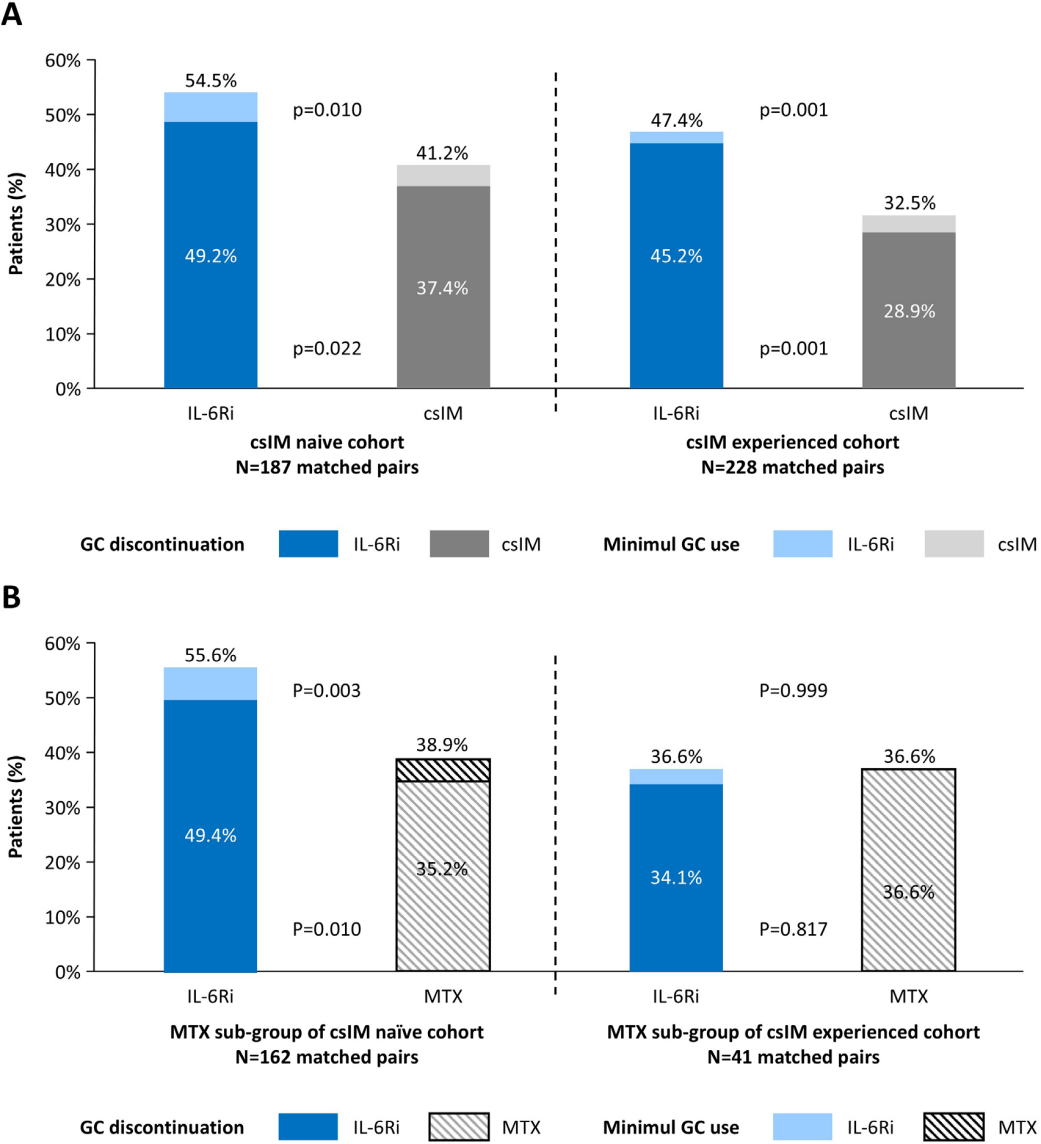
Effectiveness of primary outcomes for year 1 in PS-matched patients with PMR receiving IL-6Ri or csIM therapies in the (**A**) csIM-naïve and csIM-experienced cohorts and, (**B**) methotrexate subgroup of csIM-naïve and csIM-experienced cohorts. Minimal GC is defined as prednisone-equivalent dose ≤2 mg/day or stop GC use. Mean daily prednisone equivalents are calculated as (mg strength × quantity dispensed × conversion factor)/days supplied. The value of p is analysed by Pearson’s χ^2^ test and Wilcoxon rank-sum test. csIM, conventional synthetic immunomodulator; GC, glucocorticoid; IL-6Ri, interleukin 6 receptor inhibitor; MTX, methotrexate; PMR, polymyalgia rheumatica; PS, propensity score.

**Figure 3:**
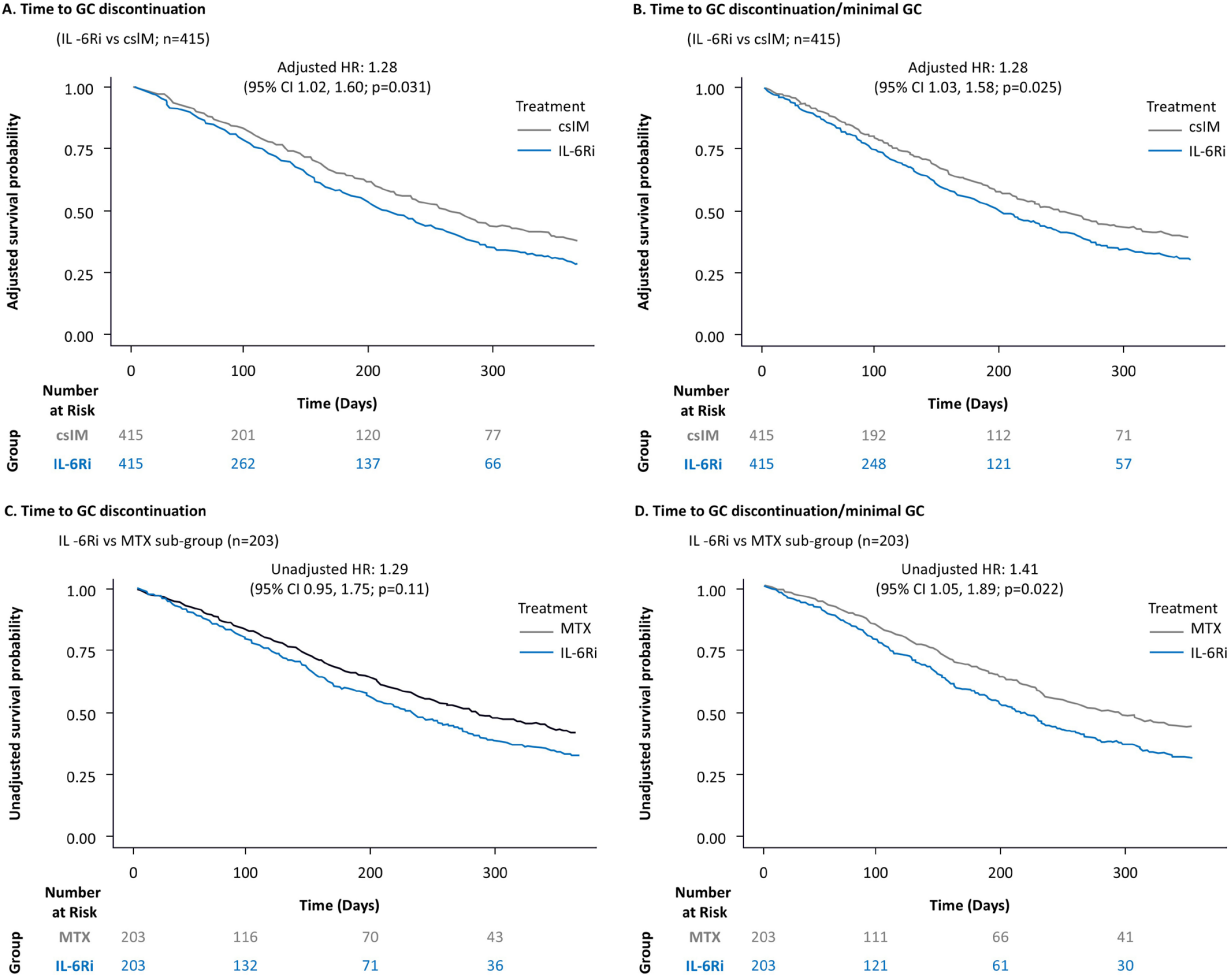
HR (95% CI)^*^ for time-to-GC discontinuation and time-to-minimal GC use after PS matching, stratified by csIM exposure for (**A–B**) IL-6Ri vs csIM^a^ and (**C–D**) IL-6Ri vs MTX^b^. ^*^For time-to-event analysis, Cox models were used to estimate HR with 95% CI. ^a^Adjusted for sex, race, region, baseline and index day PED category, calendar year of index date, Crohn’s disease, psoriasis and Charlson Comorbidity Index category. ^b^No adjustments were made as the differences were not statistically different. CI, confidence interval; csIM, conventional synthetic immunomodulator; GC, glucocorticoid; HR, hazard ratio; IL-6Ri, interleukin 6 receptor inhibitor; MTX, methotrexate; PED, prednisone-equivalent dose; PS, propensity score.

In cohort 1, a significantly higher proportion of IL-6Ri initiators discontinued GC (49.2% vs 37.4%; p=0.022) or achieved minimal GC use by year 1 than that of csIM initiators (54.5% vs 41.2%; p=0.010) (figure 2A). Similar results were found in cohort 2, that is, the proportion of IL-6Ri initiators who discontinued GC (45.2% vs 28.9%; p<0.001) or achieved minimal GC use by year 1 was higher than that of csIM initiators (47.4% vs 32.5%; p=0.001). For the primary endpoints of time-to-GC discontinuation and time-to-minimal GC use, stratified by csIM exposure, IL-6Ri initiators were significantly more likely to discontinue GC (adjusted HR (aHR) 1.28, 95% CI 1.02 to 1.60; p=0.031) or achieve the composite outcome of minimal GC use (aHR 1.28, 95% CI 1.03 to 1.58; p=0.025) by year 1 than csIM initiators (figure 3A,B), and the results remained significant up to 1 year irrespective of the censoring rules applied (online supplemental table 6).

Other than censoring for the outcome in both cohorts, there were no meaningful differences in other categories for censoring in cohort 1, whereas in cohort 2, censoring for discontinuation of index therapy was lower in the IL-6Ri group than in the csIM group (35.1% vs 51.8%) (online supplemental table 7).

In the MTX subgroup analysis, a significantly higher proportion of IL-6Ri initiators discontinued GC (49.4% vs 35.2%; p=0.010) or achieved minimal GC use (55.6% vs 38.9%; p=0.003) by year 1 in cohort 1 than that of MTX initiators, but the differences were not significant for either outcome in cohort 2 (figure 2B). IL-6Ri initiators were significantly more likely to achieve minimal GC use by year 1 than MTX initiators (HR 1.41, 95% CI 1.05 to 1.89; p=0.022). Numerically similar but non-significant differences were found between IL-6Ri and MTX initiators for discontinuation of GC (HR 1.29, 95% CI 0.95 to 1.75; p=0.11) (figure 3C,D). Time-to-GC discontinuation was evaluated separately for cohort 1 and cohort 2 in the MTX subgroup, considering the significant difference in the proportion of patients who discontinued GC or achieved minimal GC use in cohort 1 but not for time-to-GC discontinuation when stratified by csIM exposure. The results were significant for cohort 1 (HR 1.41, 95% CI 1.00 to 1.98; p=0.048), but not for cohort 2 (HR 0.84, 95% CI 0.40 to 1.75; p=0.640).

The mean (SD) weekly PED (mg per person-week) was numerically lower for IL-6Ri initiators than for csIM initiators (cohort 1: 49 (37) vs 54 (42), p=0.264; cohort 2: 47 (39) vs 50 (34) mg, p=0.107). The mean (SD) weekly PED (mg per person-week) for IL-6Ri initiators versus MTX initiators was 50 (38) and 53 (42), respectively, (p=0.543) in cohort 1, and 44 (25) and 51 (31) mg, respectively, (p=0.441) in cohort 2. In the 3-month interval analysis, the csIM group showed numerically greater reduction in GC dose in the first 3-month interval; thereafter, the IL-6Ri group showed numerically greater reduction in GC dose, which increased with each 3-month interval through year 1. During the last 3 months, the IL-6Ri group had 8.9 mg/week and 13.6 mg/week PED more reduction in GC use than csIM and MTX initiators, respectively (p=0.09 for both comparisons) (table 2; online supplemental table 8). There was a significant trend over the 1-year follow-up period showing that GC dose reduction was greater in the IL-6Ri-treated patients compared with csIM-treated patients (p=0.016).

**Table 2:** Change in weekly prednisone-equivalent dose by period after index in the IL-6Ri group, referent to csIM therapy after PS matching

Days from index	IL-6Ri N*	csIM N*	Difference in weekly GC dose (mg/week)^†^ (95% CI)	P value	P for trend
Days 1–90	415	415	1.3 (−5.3 to 8.0)	0.694	0.016
Days 91–180	411	414	−1.7 (−9.6 to 6.2)	0.675	
Days 181–270	409	406	−3.6 (−13.0 to 5.7)	0.445	
Days 271–365	386	383	−8.9 (−19.3 to 1.5)	0.093	

* Patients with available data with ≥1 day in the interval. Patients were censored for death, enrolment end − 60 days, index date + last day of assessment period, stopping index therapy, switching/adding therapy.

† A difference of 1 unit in the above table represents 1 mg change in weekly PED dose between the exposure group (IL-6Ri) and the comparator (csIM). Negative numbers reflect a greater reduction in weekly PED dose in the IL-6Ri-treated patients compared with the csIM and MTX groups.

csIM, conventional synthetic immunomodulator; GC, glucocorticoid; IL-6Ri, interleukin 6 receptor inhibitor; MTX, methotrexate; PED, prednisone-equivalent dose; PS, propensity score.

**Figure 4:**
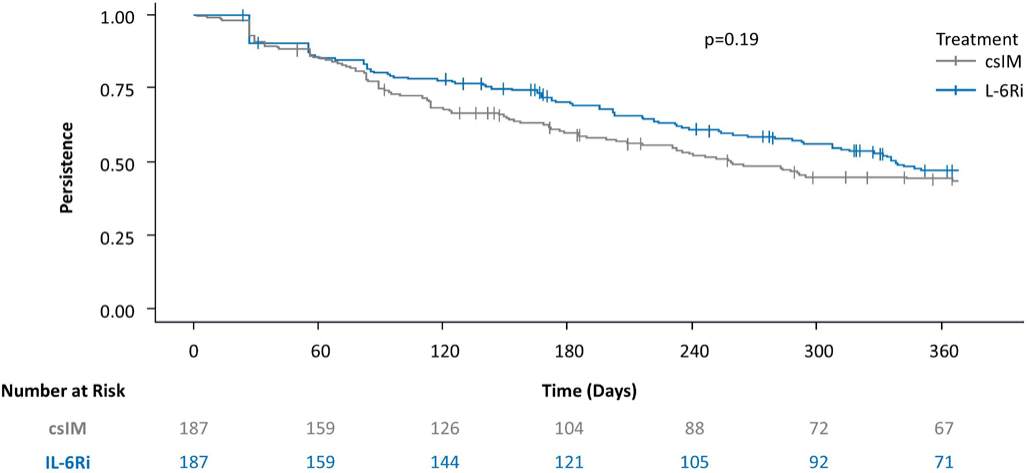
Survival curve for persistence in the csIM-naïve cohort (cohort 1) after PS matching. csIM, conventional synthetic immunomodulator (azathioprine, leflunomide, methotrexate); IL-6Ri, interleukin 6 receptor inhibitor (tocilizumab, sarilumab); PS, propensity score.

For cohort 1, persistence trended in favour of IL-6Ri, with a higher proportion of IL-6Ri initiators continuing therapy (p=0.19) (figure 4).

In the sensitivity analysis excluding patients with history of GCA, the results were consistent with the main findings. Among 451 IL-6Ri-matched and csIM-matched pairs (183 csIM-naïve and 268 csIM-experienced), IL-6Ri initiators were significantly more likely to discontinue GC at year 1 (aHR 1.31, 95% CI 1.05 to 1.62; p=0.015) or achieve the composite outcome of minimal GC use (aHR 1.30, 95% CI 1.06 to 1.60; p=0.014) than csIM initiators (online supplemental figure 1). Findings for other outcomes were also consistent with the primary findings (online supplemental tables 8 and 9).

### Safety outcomes

**Figure 5:**
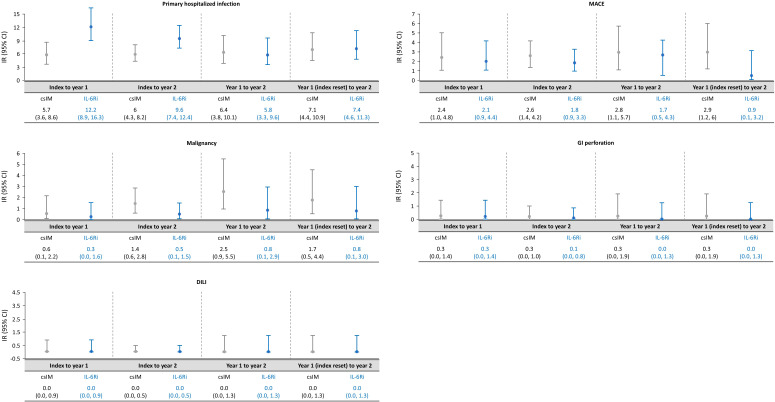
IR for safety events after PS matching in the IL-6Ri versus csIM cohort (n=415). Index to year 1 = day 1 up to day 365, Index to year 2 = day 1 up to day 730, and year 1 to year 2 = day 366 up to day 730. With the exception of primary hospitalised infections, for year 1 to year 2 without reindexing, patients with a prior event during days 1–365 were not included in the pool of at-risk patients for analysis. With reindexing, patients with a prior event during days 1–365 were reindexed at day 366 and included in the pool of at-risk patients for analysis. csIM, conventional synthetic immunomodulator; DILI, drug-induced liver injury; GI, gastrointestinal; IL-6Ri, interleukin 6 receptor inhibitor; IR, incidence rate; MACE, major adverse cardiovascular event; PS, propensity score.

The IRs of safety events evaluated in this study were similar between the groups over 2 years. Stratification of the results by the year of follow-up revealed similar IRs for all adverse events except for primary hospitalised infections in year 1, which were higher in the IL-6Ri group than in the csIM group (IR 12.2 (95% CI 8.9 to 16.3) vs 5.7 (95% CI 3.6 to 8.6), respectively). Similar IRs were observed between the groups in year 2, with or without reindexing patients at year 1. The IRs for DILI and GI perforation were low (<1 per 100 PYs) and comparable between IL-6Ri and csIM initiators (figure 5).

Overall, the HRs were not significantly different between IL-6Ri and csIM initiators over 2 years or when stratified by year for all AESI, any infection and all-cause hospitalisation. The number of hospitalised infections (any diagnosis position) was higher during year 1 in the IL-6Ri group than in the csIM group (HR 1.52, 95% CI 1.03 to 2.24) but not in year 2, with or without reindexing or over the entire 2-year period (table 3).

**Table 3:** HRs (95% CI) for adverse events of special interest for patients receiving IL-6Ri versus csIM therapy

Event name to exposure year	HR (95% CI)
All AESI (hospitalised infection primary diagnosis, GI perforation, MACE, malignancies, DILI)	
Index to year 1	1.43 (0.82 to 2.50)
Index to year 2	1.04 (0.67 to 1.63)
Year 1 to year 2*	0.54 (0.24 to 1.22)
Year 1 (index reset) to year 2^†^	0.69 (0.32 to 1.48)
Any infection	
Index to year 1	1.08 (0.91 to 1.29)
Index to year 2	1.08 (0.92 to 1.27)
Year 1 to year 2*	1.06 (0.70 to 1.59)
Year 1 (index reset) to year 2^†^	0.99 (0.80 to 1.22)
Hospitalised infections (any position diagnosis)	
Index to year 1	1.52 (1.03 to 2.24)
Index to year 2	1.24 (0.90 to 1.70)
Year 1 to year 2*	0.82 (0.47 to 1.43)
Year 1 (index reset) to year 2^†^	0.92 (0.57 to 1.47)
All-cause hospitalisation	
Index to year 1	1.10 (0.84 to 1.43)
Index to year 2	1.09 (0.87 to 1.36)
Year 1 to year 2*	1.06 (0.70 to 1.61)
Year 1 (index reset) to year 2^†^	1.00 (0.74 to 1.36)

Index to year 1 = days 1–365, index to year 2 = days 1–730.

* With the exception of primary hospitalised infections, patients with a prior event during days 1–365 were not included in the pool of at-risk patients for analysis.

† With the exception of primary hospitalised infections, patients with a prior event during days 1–365 were reindexed at day 366 and included in the pool of at-risk patients for analysis.

AESI, adverse events of special interest; csIM, conventional synthetic immunomodulator; DILI, drug-induced liver injury; GI, gastrointestinal; IL-6Ri, interleukin-6 receptor inhibitor; MACE, major adverse cardiovascular event.

## Discussion

To date, this is the largest real world study reporting on the safety and effectiveness of IL-6Ri therapy in patients with PMR. Our results showed that IL-6Ri initiators were more likely to discontinue GCs or achieve the composite outcome of minimal GC use by year 1 versus csIM initiators. The observed effectiveness of IL-6Ri therapy, based on real world data, was consistent with previously published prospective and retrospective studies on IL-6Ri therapy in patients with PMR.^21 22 29–31^ The phase III SAPHYR study on sarilumab use in relapsing PMR revealed that at week 52, 45% patients did not have any signs and symptoms of PMR and had discontinued GC.^22^ The SEMAPHORE trial of tocilizumab in steroid-refractory PMR found that 49% patients had discontinued GC by week 24.^31^ These results were consistent with the proportion of IL-6Ri initiators in the present analysis who discontinued GC by week 52, that is, 49.2% and 45.2% in the csIM-naïve and csIM-experienced cohorts, respectively. Consistent results observed in the sensitivity analysis excluding patients with a history of GCA support the observation that prior GCA status did not confound the association evaluated in this study.

Comparative analysis of IL-6Ri and MTX provided similar results in cohort 1, but not in cohort 2—potentially due to selection bias and small sample size (n=41 MTX initiators). In cohort 2, most IL-6Ri initiators had prior MTX use, while most MTX initiators had prior LEF use. Given the limited data regarding LEF use in PMR and a high prevalence of seronegative RA, prior LEF in MTX initiators may have been used for RA rather than PMR, whereas for IL-6Ri initiators, history of seronegative RA may have reflected coding bias to obtain reimbursement for off-label use of IL-6Ri, introducing confounding. Moreover, a previous study demonstrated significant reduction in IL-6 levels in MTX-treated patients with RA but not in LEF-treated patients.^32^ Thus, the IL-6 burden might have been lower in IL-6Ri initiators after MTX use than in MTX initiators after LEF use, thereby reducing the observed effect size. In accordance with these results, in cohort 2, where sample size was larger and almost all patients in both groups had prior MTX use, a significant difference in the proportion of patients who discontinued GC at year 1 was observed for IL-6Ri initiators versus csIM initiators, with 71% being LEF users (45.2% vs 28.9%; p<0.001).

Although a larger proportion of patients discontinued GC or achieved minimal GC use with IL-6Ri than with csIM or MTX therapy, significant differences were not observed in the average weekly GC dose across 3-month periods. This may be due to residual confounding because patients receiving IL-6Ri therapy appeared to have more difficult-to-treat disease. This was evidenced by numerically greater reduction in GC use in the csIM group in the first 3 months, whereas there was greater reduction in the IL-6Ri group in all subsequent 3-month periods through year 1. Similar tapering strategies in the first 3–6 months and lack of data with shorter GC taper during the study period may have masked the differences observed later. Censoring due to discontinuation of index therapy, which occurred earlier in the csIM group for cohort 1 and more frequently in csIM-experienced patients, may have also influenced the results. Large variability in GC dose may have also affected the ability to reflect a statistical difference. Despite these limitations, the differences between exposure groups increased over time, particularly in the last 3 months, when the IL-6Ri group showed numerically greater reduction in GC use (120 mg vs csIM; 183 mg vs MTX), and in the weighed regression analysis comparing IL-6Ri versus csIM initiators where the trend across periods was statistically significant.

Further, our study period largely preceded regulatory approval of IL-6Ri for PMR (sarilumab receiving US FDA approval in February 2023), which may have led to more conservative GC tapering when IL-6Ri therapies were used off-label. Consequently, inclusion of patients treated prior to formal approval may underestimate the true GC-sparing potential of IL-6Ri. Supporting this interpretation, a recent analysis presented at the 2024 American College of Rheumatology Annual Meeting evaluating postapproval IL-6Ri use in PMR demonstrated substantially greater GC reduction. In PS–matched comparisons with MTX-exposed patients, sarilumab-treated patients achieved approximately 45% lower GC utilisation at 10 months, despite relatively small cohort sizes (p=0.03).^33^

Larger sample size and longer follow-up may be needed to more completely demonstrate the steroid-sparing benefit of IL-6Ri therapies based on when patients more aggressively taper, which may be beyond 6–12 months rather than earlier as occurred in PMR clinical trials of IL-6Ri.

Several European rheumatology society guidelines recommend targeting a reduction to 10 mg/day of prednisone (or equivalent) within 4–8 weeks, followed by tapering by approximately 1 mg every 4 weeks, with the goal of discontinuation within 1 year of treatment initiation.^25 26^ In clinical practice, patients who experience repeated failures of tapering attempts, or in whom GC-related toxicities become a limiting factor, often represent a scenario where the benefit–risk profile increasingly favours the earlier introduction of biologic GC sparing therapy.

A recent study reported that 52% of patients with PMR continued tocilizumab at 24 months, indicating that some patients may require extended treatment.^30^ However, treatment in randomised controlled trials of IL-6Ri therapies was limited to 4–12 months, had small sample sizes and lacked open-label extension studies, making it difficult to evaluate long-term safety.

This study provides important information about the safety of IL-6Ri therapy in PMR. We found comparable risk of the evaluated safety events for the IL-6Ri and csIM therapy groups over a 2-year follow-up period. A higher incidence of primary hospitalised infections and all hospitalised infections was found in the IL-6Ri group during year 1, with the relative risk (RR) between groups being consistent with that found in randomised controlled trials in patients with RA. The HR (95% CI) for all hospitalised infections in the current study was 1.52 (95% CI 1.03 to 2.24); the RR for primary hospitalised infections was 2.14 (12.2 (IL-6Ri) vs 5.7 (csIM) per 100 PY), which was similar to the RR of serious infections in RA with sarilumab 200 mg plus disease-modifying antirheumatic drugs (DMARDs) versus DMARDs (RR: 1.39), with small differences in the absolute risks: 4.3 and 3.1 events per 100 PY, respectively,^34^ as well as to the RR of tocilizumab 8 mg/kg plus DMARDs versus DMARDs (RR: 1.36; 5.3 and 3.9 per 100 PY), respectively.^35^ The higher rate found in the IL-6Ri group may have also reflected selection bias if patients at higher risk of infection were more likely to receive IL-6Ri therapy. Overall, these results are consistent with the established safety profile of IL-6Ri therapies.^19–21 29 30 36 37^ The risk of hospitalised infections during the first year should be weighed against the benefit of reducing long-term GC exposure and the potential for other GC-related adverse events.

We also found that the overall IR of primary hospitalised infections was higher in both groups than the IR of serious infections observed in randomised controlled trials of RA. This is consistent with the observations made for tocilizumab in combination with a GC taper in GCA, a disease sometimes associated with PMR. The observed rates are also consistent with a French-population study, which found severe infection incidence of 11.1/100 PYs (95% CI 8.3 to 14.6) in patients with GCA.^38^ Although patients with GCA may receive higher GC doses, the incidence of serious infections in patients with PMR and GCA is similar, that is, 17.7 and 18.9 per 100 US inpatient hospitalisation claims, respectively.^14^ The older age of patients with PMR and GC use may increase the risk of infections, potentially contributing to higher overall IRs compared with patients with RA. Differences in patient populations—such as healthier individuals typically enrolled in clinical trials versus real world patients—and selection bias may also have influenced these findings. Furthermore, patients at elevated risk of infection may be more likely to receive steroid-sparing therapies to minimise GC exposure.

One potential limitation of this study is the inclusion of patients with a history of seronegative RA, based on the assumption that they could meet classification criteria for both conditions and may be initially misdiagnosed. PS matching was used to control for this condition; however, the diagnosis of seronegative RA may have been used to obtain reimbursement for off-label use of IL-6Ri therapy, which is less likely for MTX due to its low cost. Although seronegative arthritis may have been more prevalent in the IL-6Ri cohort, this might be expected to bias results conservatively towards the csIM group, as seronegative disease may be associated with a lower likelihood of GC discontinuation. Particularly in the csIM-experienced patients in cohort 2, where nearly 70% of patients had a prior diagnosis of seronegative RA, it is unclear whether it reflects effectiveness for RA rather than PMR. However, the effect size was similar in the csIM-naïve patients in cohort 1, where the incidence of seronegative RA was lower (~30%), suggesting greater effectiveness for IL-6Ri therapy versus csIM therapy or MTX alone in patients with PMR.

Administrative health insurance claims data are prone to incomplete or inaccurate diagnoses codes, which could affect inclusion and exclusion criteria. However, the use of administrative claims data to identify patients with PMR has high sensitivity and specificity.^39^ Medication use was inferred from prescription patterns, which, despite validation, could lead to misclassification of GC dose and residual confounding, particularly as patients taper GC therapy.^40^ However, such misclassification is unlikely to vary by exposure status. In addition, GC discontinuation was used as a surrogate for effectiveness and could possibly be dependent on both providers’ and patients’ motivation to discontinue GC.

Finally, other residual confounding factors such as socioeconomic status, laboratory or clinical findings could not be analysed or adjusted for, due to the unavailability of data, and they may have influenced medication decisions. We recognise that all IL-6Ri use for PMR included in this analysis may have been off-label (given sarilumab was approved in 2023 for PMR and tocilizumab approved only for GCA in 2017); thus, IL-6Ri-treated patients were likely to have experienced more severe disease, making our results subject to residual confounding by disease severity. Therefore, our results may reflect conservative estimates.

Beyond the limitations of claims data, other factors may affect the generalisability of these results. The data represent a Medicare-age population, where the majority is aged ≥65 years, potentially underrepresenting younger patients with PMR. Additionally, the data primarily represent tocilizumab treatment in the IL-6Ri group. Furthermore, the requirement of only 180-day baseline enrolment may not have provided enough observation time to control for potential confounders, such as prior GC therapy, csIM therapy or time since the first documented PMR diagnosis.

## Conclusions

To our knowledge, this is the first study directly comparing IL-6Ri therapy with csIM or MTX therapy. It addresses an important research gap and provides additional support for recent society recommendations that favour IL-6Ri over MTX in steroid-dependent PMR and in patients with major comorbidities. In this real world study, IL-6Ri therapy was more effective than csIM therapy (overall and MTX specifically) in discontinuing or minimising GC use at year 1 in csIM-naïve and csIM-experienced patients with PMR, with safety being consistent with results of previous studies. Thus, IL-6Ri therapy should be considered as an important option for patients with GC-dependent PMR.

## Supplementary material

10.1136/rmdopen-2026-006850 online supplemental file 1

## Data Availability

All data relevant to the study are included in the article or uploaded as supplementary information.
