# Supplementary material for: Effectiveness and safety of interleukin 6 receptor inhibitors versus conventional immunomodulatory therapy in steroid-refractory polymyalgia rheumatica
Source: RMD Open. 2026 Aug 3;12(3):e006850. doi: 10.1136/rmdopen-2026-006850 (PMC13435999; doi:10.1136/rmdopen-2026-006850)
Supplement: Supplementary file 1 [file rmdopen-12-3-s001.docx]

Supplemental material

[Supplemental Table 1: Patient attrition	2](#_Toc228260670)

[Supplemental Table 2. Definitions of baseline exclusion measures	3](#_Toc228260671)

[Supplemental Table 3: Definitions of effectiveness and safety variables	4](#_Toc228260672)

[Supplemental Table 4. Characteristics of patients with PMR receiving IL-6Ri or csIM in the csIM-naïve or csIM-experienced cohorts before PS-matching	7](#_Toc228260673)

[Supplemental Table 5. Characteristics of patients with PMR receiving IL-6Ri or MTX in the MTX subgroup of csIM-naïve or csIM-experienced cohort after PS-matching	9](#_Toc228260674)

[Supplemental Table 6: Effectiveness primary outcomes by varying censoring rules in the IL-6Ri versus csIM and MTX subgroup	11](#_Toc228260675)

[Supplemental Table 7. Reason for end of follow-up for discontinuation of GC endpoint after PS-matching	12](#_Toc228260676)

[Supplemental Table 8: Change in weekly prednisone-equivalent dose by duration from the index date in the IL-6Ri group, referent to csIM for MTX subgroup and sensitivity analysis (excluding GCA history) after PS-matching	13](#_Toc228260677)

[Supplemental Table 9. Outcomes at year 1 for PS-matched patients included in the sensitivity analysis (excluding patients with history of GCA) and receiving IL-6Ri or csIM therapies in the csIM-naïve or csIM-experienced cohort	14](#_Toc228260678)

[SUPPLEMENTAL FIGURE 1: HR^a^ (95% CI) for patients on IL-6Ri versus csIM therapy included in the sensitivity analysis (excluding patients with history of GCA before direct-matching) after PS-matching	15](#_Toc228260679)

Supplemental Table 1: Patient attrition

|  | csIM-naïve cohort  (Cohort 1) | | csIM-experienced cohort  (Cohort 2) | |
| --- | --- | --- | --- | --- |
| Criteria, n | **IL-6Ri** | **csIM** | **IL-6Ri** | **csIM** |
| IL-6Ri patient initiation date range: 2006–2020 | 57,788 | 1,183,190 | 57,788 | 1,183,190 |
| Initiation after 03/29/2016 | 31,957 | 443,886 | 31,957 | 443,886 |
| Initiation on or before 06/30/2020 | 28,587 | 406,287 | 28,587 | 406,287 |
| Meet PMR definition on or before index date | 2,639 | 18,242 | 2,639 | 18,242 |
| With oral GC prescription spanning the index date | 1,472 | 12,213 | 1,472 | 12,213 |
| With ≥180 days baseline and ≥1 day follow-up | 1,298 | 9,814 | 1,298 | 9,814 |
| Age ≥18 years on index date | 1,298 | 9,814 | 1,298 | 9,814 |
| With daily PED ≤25 mg | 940 | 8,616 | 940 | 8,616 |
| Without seropositive RA at baseline | 764 | 7,417 | 764 | 7,417 |
| Without other inflammatory arthritis, MS or connective tissue disease or solid organ transplant at baseline | 690 | 6,416 | 690 | 6,416 |
| No active treatment for malignancy at baseline | 668 | 6,281 | 668 | 6,281 |
| Did not initiate csIM and IL-6Ri on same day | 663 | 6,281 | 663 | 6,281 |
| No prior IL-6Ri use | 663 | 6,210 | 663 | 6,210 |
| Age ≥50 years | 663 | 6,200 | 663 | 6,200 |
| No prior csIM (csIM-naïve)/Had prior csIM (csIM-experienced) | 251 | 5,505 | 412 | 991 |
| Direct-matching^a^ (3:1 csIM to IL-6Ri) | 187 | 502 | 228 | 463 |
| PS-matching^b^ (1:1 csIM to IL-6Ri) | 187 | 187 | 228 | 228 |
| PS-matched pairs with MTX as the csIM | 162 | 162 | 41 | 41 |

^a^csIM initiators were direct-matched to IL-6Ri initiators (up to 3:1) based on age (±3 years), sex, any history of GCA, baseline period daily GC dose [7 categories from <2.5 mg to >25.0 mg PED], daily GC dose at index [6 categories: <2.5 mg to 20.0–25.0 mg PED], and temporal proximity of prior csIM therapy (time from prior csIM use to index; 1–60, 61–180, >180 days [in Cohort 2 only]).

^b^Additional PS-matching covariates included race, region, calendar year of index treatment, reason for entering Medicare, Charlson Comorbidity Index (CCI) category, time from first PMR diagnosis to index, baseline healthcare resource utilization (number of inpatient days, emergency department visits, outpatient visits), conditions that may be associated with GC use (asthma, atopic dermatitis, chronic obstructive pulmonary disease, Crohn’s disease, psoriasis, ulcerative colitis), and seronegative RA.

csIM, conventional synthetic immunomodulator; GC, glucocorticoid; IL-6Ri, interleukin-6 receptor inhibitors; MS, multiple sclerosis; MTX, methotrexate; PED, prednisone-equivalent dose; PMR, polymyalgia rheumatica; PS, propensity score; RA, rheumatoid arthritis.

# Supplemental Table 2. Definitions of baseline exclusion measures

| **Exclusion Measure** | **ICD-10-CM code list** |
| --- | --- |
| ANCA-associated vasculitis  (Component conditions : Microscopic polyangiitis, Wegener's granulomatosis, Churg-Strauss syndrome) | M31.7, M31.3*, M30.1 |
| Ankylosing spondylitis | M45.x, M08.1 |
| Bechet’s disease | M35.2 |
| Dermatomyositis | M33.0x, M33.1x, M33.9x |
| Mixed connective tissue disease | M35.1, M35.89, M35.9 |
| Multiple sclerosis | G35.* |
| Other spondyloarthritides | M46.x |
| Organ transplantation | Z94.* |
| Polymyositis | M33.2x |
| Psoriatic arthritis | L40.5x |
| Scleroderma | L94.0, L94.1, M34.x |
| Sjogren’s syndrome | M35.0x |
| Systemic lupus erythematosus | M32.x |
| Seropositive RA | M05 M061/*7140 7142 71481* |
| Takayasu's arteritis | M31.4 |
| Uveitis | H44.11*, H44.13* |

ANCA, antineutrophil cytoplasmic antibody; ICD-10-CM, international classification of diseases-10th revision-clinical modification; RA, rheumatoid arthritis.

# Supplemental Table 3: Definitions of effectiveness and safety variables

| Variable | Definition | Measure type^#^ |
| --- | --- | --- |
| Treatment^a^ | | |
| IL-6Ri use | Receipt of a prescription or medical claim for NDC generic name of “tocilizumab” or “sarilumab”  OR a medical services claim with Current Procedural Terminology (CPT) or Healthcare Common Procedure Coding System (HCPCS) code of C9264 or J3262 | Dichotomous  [n (%)] |
| csIM use | Receipt of a prescription or medical claim with NDC generic name of “methotrexate,” “leflunomide,” or “azathioprine”  OR a medical services claim with CPT/HCPCS code of J8610, J9250, J9260, J7500, or J7501 | Dichotomous  [n (%)] |
| Demographic characteristics | | |
| Age | Age in years on index date | Continuous [years] |
| Age category | Age in years category on index date | Categorical [years]  [50–64, 65–74, 75–84, >85] |
| Sex | Patient sex as recorded on index date | Categorical [Female, male, other/missing] |
| Race/ethnicity | Patient race/ethnicity as recorded on index date | Categorical [White, Black, Asian, Hispanic, unknown/missing] |
| Region | US Census region of patient residence on index date | Categorical [Northeast, Midwest, South, West] |
| Original reason for Medicare eligibility | Original reason for eligibility, regardless of the current reason for eligibility | Categorical [age, disability, ESRD] |
| Clinical characteristics | | |
| Charlson Comorbidity Index (CCI) Score | CCI score assessed over baseline period starting 180 days prior through 1 day prior to the index date. Individual CCI condition components with score values include:  Myocardial infarction (Score=1)  Congestive heart failure (Score=1)  Peripheral vascular disease (Score=1)  Cerebrovascular disease (Score=1)  Dementia (Score=1)  Chronic pulmonary disease (Score=1)  Rheumatic disease (Score=1)  Peptic ulcer disease (Score=1)  Mild liver disease (Score=1)  Diabetes without chronic complications (Score=1)  Diabetes with chronic complications (Score=2)  Hemiplegia or paraplegia (Score=2)  Renal disease (Score=2)  Any malignancy, including lymphoma and leukemia, except malignant neoplasm of skin (Score=2)  Moderate or severe liver disease (Score=3)  Metastatic solid tumor (Score=6)  AIDS/HIV (Score=6) | Continuous [score] |
| CCI category | Category of CCI score as defined above | Categorical [CCI score]  [0–1, 2–3, ≥4] |
| Time from PMR diagnosis to index date | Time, in days, from first recorded PMR diagnosis occurring during all available time prior to the index date | Continuous [days] |
| Duration of baseline GC therapy | Days during the baseline period starting 180 days prior to through 1 day prior to the index date on which any oral GC therapy was administered | Continuous [days] |
| Baseline length | Using all available data, days enrolled prior to and including the index date | Continuous [days] |
| Follow-up length | Using all available data, days of follow-up observation time contributed defined as the period 1 day after the index date until the earliest occurrence of disenrollment, death, discontinuation of index PMR therapy, or addition of the comparator PMR therapy type | Continuous [days] |
| Baseline daily GC dose category | Prednisone equivalents of oral GC therapy averaged during the 180-day period prior to index  Sum of person-day prednisone-equivalent dose over the 180-day period prior to index /180  Person-day prednisone-equivalent dose calculated as (Prednisone equivalents dispensed/days’ supply for each prescription, then summed for multiple prescriptions on same day) | Categorical  [mg prednisone equivalents/day] |
| Dose of oral GC therapy on index date | Daily prednisone equivalents of oral GC therapy assessed on the index date  Person-day prednisone-equivalent dose defined above on the index day | Continuous, Categorical [mg prednisone equivalents/day] |
| Temporal proximity of prior csIM therapy | Days prior to the index date using all available data, through 1 day prior to the index date on which the last csIM therapy occurred | Categorical [days]  [1–60, 61–180, >180] |
| GCA with or without mention of PMR | At least 1 inpatient or 2 outpatient claims (separated by 30 or more days and occurring within 365 days) using all available data and where GC was initiated within 30 days after 1 inpatient claim or between 1st outpatient claim and 30 days after 2nd outpatient claim at initial daily prednisone-equivalent dose of ≥40 mg  ICD-10-CM: M31.6 [GCA without PMR] or M31.5 [GCA with PMR] or ICD-9-CM:446.5 | Dichotomous |
| Asthma | Any inpatient or outpatient diagnosis (any position) during the baseline period starting 180 days prior through the index date of: ICD-10-CM: J45* | Dichotomous |
| Atopic dermatitis | Any inpatient or outpatient diagnosis (any position) during the baseline period starting 180 days prior through the index date of: ICD-10-CM: L20* | Dichotomous |
| Chronic obstructive pulmonary disease (COPD) | Any inpatient or outpatient diagnosis (any position) during the baseline period starting 180 days prior through the index date of: ICD-10-CM: J41.1, J41.8, J43, J43.2, J43.8, J44, J44.0, J44.1, J41, J41.0, J42, J43.0, J43.1, J43.9, J44.9 | Dichotomous |
| Crohn’s disease | Any inpatient or outpatient diagnosis (any position) during the baseline period starting 180 days prior through the index date of: ICD-10-CM: K50* | Dichotomous |
| Psoriasis | Any inpatient or outpatient diagnosis (any position) during the baseline period starting 180 days prior through the index date of: ICD-10-CM: L40.0, L40.1, L40.2, L40.3, L40.4, L40.8, L40.9 | Dichotomous |
| Seronegative rheumatoid arthritis | Any inpatient or outpatient diagnosis (any position) during the baseline period starting 180 days prior through index date of: ICD-10-CM: M06* (except for M06.1) | Dichotomous |
| Ulcerative colitis | Any inpatient or outpatient diagnosis (any position) during the baseline period starting 180 days prior through the index date of: ICD-10-CM: K51* | Dichotomous |
| Baseline healthcare resource utilization | | |
| Inpatient days | Sum of the length of stay of all-cause inpatient events occurring during the baseline period from 180 days prior to through 1 day prior to the index date. Inpatient events defined as medical services claim where type of service=FAC_IP.ACUTE | Continuous [days] |
| Emergency department (ED) visit | Count of discrete all-cause emergency department visits, occurring during the baseline period from 180 days prior to through 1 day prior to the index date. ED visits defined as a medical services claim where type of service=FAC_OP.ER, PROF.ER, FAC_OP.ED, PROF.ED and inpatient confinement claim=NO | Continuous/count |
| Outpatient office visit | Count of discrete all-cause, all-specialty outpatient office visits, occurring during the baseline period from 180 days prior to through 1 day prior to the index date. Outpatient office visits defined as a medical service claim where type of service=PROF.OFFVIS and inpatient confinement claim=NO | Continuous/count |
| Calendar year | Calendar year of index date | Categorical |
| Safety variables | | |
| Hospitalized infection (primary) | Presence of a) inpatient discharge with diagnosis (primary) of bacterial, viral, fungal, or parasitic infection and b) inpatient or outpatient anti-infection medication (ICD9/ICD10) | Events/100 patient-years |
| GI perforations | Presence of an inpatient discharge with diagnosis (primary or secondary) of GI perforation (ICD9/ICD10) | Events/100 patient-years |
| MACE | Presence of inpatient discharge with diagnosis (primary or secondary) of myocardial infarction and (overnight stay or died the same day), or presence of inpatient discharge (primary or secondary) of stroke, or coronary artery bypass surgery (CABG) (ICD9/ICD10, CPT) | Events/100 patient-years |
| Malignancies (excluding NMSC) | a) 1 inpatient primary discharge diagnosis code, or b) 2 or more outpatient diagnosis code from oncologist visit with [30–365] days apart from each other, or c) 1 or more outpatient diagnosis codes from oncologist visit with treatment [0–90] days before or after the malignancy diagnosis code (ICD9/ICD10, HCPCS/CPT, NDC) | Events/100 patient-years |
| DILI | Presence of inpatient discharge with diagnosis (primary or secondary) of DILI (ICD9/ICD10) | Events/100 patient-years |
| Hospitalized infection (Primary or secondary) | Presence of a) inpatient discharge with diagnosis (primary or secondary) for bacterial, viral, fungal, or parasitic infection (ICD9/ICD10) | Events/100 patient-years |
| Any infection | Any primary or secondary hospitalized infection as defined above or presence of an outpatient claim with diagnosis for a bacterial, viral, fungal, or parasitic infection or prescription for anti-infective medication (ICD9/ICD10, HCPCS/CPT, NDC) | Events/100 patient-years |
| All-cause hospitalization | Presence of any inpatient discharge | Events/100 patient-years |

^a^Prescription claims with quantity dispensed being ≤0 or days supplied for being ≤1 or >365 were excluded from analyses. Overlapping prescription claims for the same oral GC generic drug name at the same strength were assumed to be used sequentially. Sequential use is defined as shifting forward the second overlapping fill to begin immediately after the first fill’s supply ends (For GC, maximum shift was 30 days forward). Different strengths of the same generic drug were assumed to be used concurrently (e.g., simultaneous use of prednisone 5 mg and prednisone 1 mg x 2 = prednisone 7 mg/day). Oral steroids with a different generic name were assumed to replace the prior oral steroid. Overlapping prescription claims for the same oral or injectable csIM therapy with the same dose and route of administration were adjusted by shifting forward the second overlapping fill to begin immediately after the first fill’s supply ended (maximum shift 14 days forward). For overlapping fills of the same csIM therapy, where the route of administration or dose changed, it was assumed that the prior route of administration or dose was discontinued and replaced with the new route of administration or dose. For overlapping fills of IL-6Ri therapy, the second prescription immediately replaced the first prescription. No forward shift was allowed.

**^#^**Dichotomous covariates with no recorded events during the baseline period were excluded from PS models.

AIDS, acquired immunodeficiency syndrome; csIM, conventional synthetic immunomodulatory therapy; DILI, drug-induced liver injury; ESRD, end-stage renal disease; GC, glucocorticoids; GCA, giant cell arteritis; GI, gastrointestinal; HCPCS, healthcare common procedure coding system; HIV, human immunodeficiency virus; ICD, International Classification of Diseases; IL-6Ri, interleukin-6 receptor inhibitor; MACE, major adverse cardiovascular events; NDC, national drug code; NMSC, nonmelanoma skin cancer; PMR, polymyalgia rheumatica; US, United States.

# Supplemental Table 4. Characteristics of patients with PMR receiving IL-6Ri or csIM in the csIM-naïve or csIM-experienced cohorts before PS-matching

| Characteristics | csIM-naïve cohort (Cohort 1) | | | csIM-experienced cohort (Cohort 2) | | |
| --- | --- | --- | --- | --- | --- | --- |
|  | **IL-6Ri**  **N=187** | **csIM**  **N=502** | **SMD** | **IL-6Ri**  **N=228** | **csIM**  **N=463** | **SMD** |
| Temporal proximity of prior csIM use,  n (%) |  |  |  |  |  | 0.06 |
| 1–60 |  |  |  | 92 (40.4%) | 179 (38.7%) |  |
| 61–180 |  |  |  | 51 (22.4%) | 98 (21.2%) |  |
| >180 |  |  |  | 85 (37.3%) | 186 (40.2%) |  |
| Age in years at index, Mean (SD) | 75.7 (6.8) | 75.6 (6.5) | 0.00 | 74.0 (5.9) | 74.6 (5.3) | 0.10 |
| Sex, Female, n (%) | 136 (72.7%) | 366 (72.9%) | 0.00 | 162 (71.1%) | 340 (73.4%) | 0.05 |
| Baseline GC dose (mg/day) |  |  | 0.17 |  |  | 0.13 |
| <2.5 | 13 (7.0%) | 39 (7.8%) |  | 24 (10.5%) | 53 (11.4%) |  |
| 2.5–<5.0 | 24 (12.8%) | 72 (14.3%) |  | 54 (23.7%) | 125 (27.0%) |  |
| 5–<10 | 65 (34.8%) | 184 (36.7%) |  | 109 (47.8%) | 215 (46.4%) |  |
| 10–<15 | 51 (27.3%) | 144 (28.7%) |  | 30 (13.2%) | 57 (12.3%) |  |
| 15–<20 | 19 (10.2%) | 41 (8.2%) |  | <11 | Redacted |  |
| 20–25 | <11 | <11 |  | <11 | <11 |  |
| >25 | <11 | Redacted |  | 0 (0.0%) | 0 (0.0%) |  |
| Index daily GC dose category (mg), n (%) |  |  | 0.06 |  |  | 0.15 |
| <2.5 | <11 | <11 |  | <11 | 11 (2.4%) |  |
| 2.5–<5.0 | Redacted | Redacted |  | Redacted | 35 (7.6%) |  |
| 5–<10 | 45 (24.1%) | 127 (25.3%) |  | 80 (35.1%) | 192 (41.5%) |  |
| 10–<15 | 44 (23.5%) | 122 (24.3%) |  | 59 (25.9%) | 118 (25.5%) |  |
| 15–<20 | 43 (23.0%) | 110 (21.9%) |  | 31 (13.6%) | 51 (11.0%) |  |
| 20–25 | 33 (17.6%) | 81 (16.1%) |  | 31 (13.6%) | 56 (12.1%) |  |
| GCA | 26 (13.9%) | 35 (7.0%) | 0.23 | <11 | <11 | 0.10 |
| Race |  |  | 0.15 |  |  | 0.13 |
| White | 161 (86.1%) | 456 (90.8%) |  | 212 (93.0%) | 421 (90.9%) |  |
| Black | <11 | 14 (2.8%) |  | <11 | 16 (3.5%) |  |
| Other (Asian, Hispanic, unknown/ missing) | Redacted | 32 (6.4%) |  | <11 | 26 (5.6%) |  |
| Region |  |  | 0.44 |  |  | 0.19 |
| West | 45 (24.1%) | Redacted |  | 47 (20.6%) | Redacted |  |
| Midwest | Redacted | 129 (25.7%) |  | 53 (23.2%) | 129 (27.9%) |  |
| Northeast | 60 (32.1%) | 111 (22.1%) |  | 56 (24.6%) | 90 (19.4%) |  |
| South | 62 (33.2%) | 168 (33.5%) |  | 72 (31.6%) | 159 (34.3%) |  |
| Unknown | <11 | <11 |  | 0 (0.0%) | <11 |  |
| Original reason for Medicare eligibility |  |  | 0.08 |  |  | 0.04 |
| Age ≥65 years | 166 (88.8%) | 458 (91.2%) |  | 194 (85.1%) | 401 (86.6%) |  |
| Disabled | 21 (11.2%) | 44 (8.8%) |  | 34 (14.9%) | 62 (13.4%) |  |
| Charlson comorbidity index category, n (%) |  |  | 0.11 |  |  | 0.07 |
| 0–1 | 82 (43.9%) | 230 (45.8%) |  | 97 (42.5%) | 182 (39.3%) |  |
| 2–3 | 68 (36.4%) | 157 (31.3%) |  | 87 (38.2%) | 183 (39.5%) |  |
| ≥4 | 37 (19.8%) | 115 (22.9%) |  | 44 (19.3%) | 98 (21.2%) |  |
| Time from first PMR diagnosis to index date, Median (IQR) | 295.0  (133.0, 717.5) | 247.0  (112.3, 574.0) | 0.19 | 704.0 (278.8, 1,438.5) | 598.0 (273.0, 1,262.5) | 0.07 |
| Baseline inflammatory conditions, n (%) |  |  |  |  |  |  |
| Asthma | 16 (8.6%) | 58 (11.6%) | 0.10 | 26 (11.4%) | 37 (8.0%) | 0.12 |
| Atopic dermatitis | <11 | <11 | 0.05 | <11 | <11 | 0.02 |
| COPD | 16 (8.6%) | 59 (11.8%) | 0.11 | 17 (7.5%) | 58 (12.5%) | 0.17 |
| Crohn’s disease | 0 (0.0%) | <11 | 0.11 | <11 | <11 | 0.02 |
| Psoriasis | <11 | 14 (2.8%) | 0.18 | <11 | <11 | 0.00 |
| UC | 0 | <11 | 0.11 | <11 | <11 | 0.06 |
| Seronegative RA | 59 (31.6%) | 121 (24.1%) | 0.17 | 160 (70.2%) | 252 (54.4%) | 0.33 |
| Baseline number of inpatient days |  |  | 0.11 |  |  | 0.02 |
| Mean (SD) | 1.1 (4.4) | 0.7 (2.5) |  | 0.7 (2.9) | 0.7 (2.3) |  |
| Median (IQR) | 0.0 (0.0, 0.0) | 0.0 (0.0, 0.0) |  | 0.0 (0.0, 0.0) | 0.0 (0.0, 0.0) |  |
| Baseline number of ED visits |  |  | 0.07 |  |  | 0.09 |
| Mean (SD) | 0.4 (1.1) | 0.5 (1.1) |  | 0.4 (0.9) | 0.5 (1.1) |  |
| Median (IQR) | 0.0 (0.0, 0.5) | 0.0 (0.0, 1.0) |  | 0.0 (0.0, 0.0) | 0.0 (0.0, 0.0) |  |
| Baseline number of outpatient office visits |  |  | 0.12 |  |  | 0.11 |
| Mean (SD) | 10.3 (5.7) | 9.6 (5.3) |  | 9.9 (5.3) | 9.4 (5.0) |  |
| Median (IQR) | 9.0 (6.0, 13.0) | 9.0 (6.0, 13.0) |  | 9.0  (6.0, 12.0) | 8.0 (6.0, 12.0) |  |
| Calendar year of index date |  |  | 0.29 |  |  | 0.29 |
| 2016 | <11 | 68 (13.5%) |  | 27 (11.8%) | 71 (15.3%) |  |
| 2017 | 41 (21.9%) | 103 (20.5%) |  | 71 (31.1%) | 95 (20.5%) |  |
| 2018 | 55 (29.4%) | 144 (28.7%) |  | 65 (28.5%) | 126 (27.2%) |  |
| 2019 | 56 (29.9%) | 131 (26.1%) |  | 50 (21.9%) | 120 (25.9%) |  |
| 2020 | Redacted | 56 (11.2%) |  | 15 (6.6%) | 51 (11.0%) |  |

To protect patient privacy and prevent potential identification, results are reported only when the number of patients is ≥11. Additionally, data are redacted in cases where reporting results for ≥11 patients could enable derivation of counts <11.

AD, atopic dermatitis; COPD, chronic obstructive pulmonary disease; csIM, conventional synthetic immunomodulatory therapy; ED, emergency department; GC, glucocorticoid; GCA, giant cell arteritis; IL-6Ri, interleukin-6 receptor inhibitor; IQR, interquartile range; PMR, polymyalgia rheumatica; PS, propensity score; RA, rheumatoid arthritis; SD, standard deviation; SMD, standardized mean difference; UC, ulcerative colitis.

# Supplemental Table 5. Characteristics of patients with PMR receiving IL-6Ri or MTX in the MTX subgroup of csIM-naïve or csIM-experienced cohort after PS-matching

| **Characteristics** | **MTX sub-analysis of csIM-naïve cohort** | | | | **MTX sub-analysis of csIM-experienced cohort** | | | | |
| --- | --- | --- | --- | --- | --- | --- | --- | --- | --- |
|  | **IL-6Ri**  **(N=162)^a^** | **MTX**  **(N=162)^a^** | **SMD** | **p-value^b^** | **IL-6Ri**  **(N=41)^a^** | **MTX**  **(N = 41)^a^** | | **SMD** | **p-value^b^** |
| **No. of prior csIM,**  **mean (SD)** | **-** | **-** | **-** | **-** | 1.2 (0.4) | 1.0 (0.0) | | **-** | 0.006 |
| **Prior csIM,**  **n (%)** |  |  |  |  |  |  | |  |  |
| Methotrexate | **-** | **-** | **-** | **-** | 38 (92.7%) | 0 (0.0%) | | **-** | <0.001 |
| Leflunomide | **-** | **-** | **-** | **-** | <11 | 37 (90.2%) | | **-** | <0.001 |
| Azathioprine | **-** | **-** | **-** | **-** | 0 (0.0%) | <11 | | **-** | 0.116 |
| **Temporal proximity of prior csIM use, n (%)** |  |  |  |  |  |  | | 0.53 | 0.071^3^ |
| 1–60 days | **-** | **-** | **-** | **-** | 18 (43.9%) | 20 (48.8%) | |  |  |
| 61–180 days | **-** | **-** | **-** | **-** | <11 | <11 | |  |  |
| >180 days | **-** | **-** | **-** | **-** | Redacted | Redacted | |  |  |
| **Age in years at index date** |  |  | 0.01 | 0.835^1^ |  |  | | 0.06 | 0.780^1^ |
| Mean (SD) | 75.8 (7.0) | 75.7 (6.0) |  |  | 75.0 (6.8) | 74.7 (4.7) | |  |  |
| **Sex** |  |  | 0.00 | >0.999^3^ |  |  | | 0.27 | 0.230^3^ |
| Female, n (%) | 118 (72.8%) | 118 (72.8%) |  |  | 26 (63.4%) | 31 (75.6%) | |  |  |
| **Baseline daily GC dose category (mg), n (%)** |  |  | 0.11 | 0.968^2,3^ |  |  | | 0.34 | 0.715^2,3^ |
| <2.5 | <11 | 11 (6.8%) |  |  | <11 | <11 | |  |  |
| 2.5–<5.0 | 18 (11.1%) | 22 (13.6%) |  |  | <11 | <11 | |  |  |
| 5–<10 | 62 (38.3%) | 61 (37.7%) |  |  | 21 (51.2%) | 24 (58.5%) | |  |  |
| 10–<15 | 43 (26.5%) | 39 (24.1%) |  |  | <11 | <11 | |  |  |
| 15–<20 | 16 (9.9%) | 14 (8.6%) |  |  | <11 | <11 | |  |  |
| 20–25 | <11 | <11 |  |  | 0 (0.0%) | 0 (0.0%) | |  |  |
| >25 | <11 | <11 |  |  | 0 (0.0%) | 0 (0.0%) | |  |  |
| **Index day GC dose category (mg), n (%)** |  |  | 0.11 | 0.964^2^ |  |  | | 0.47 | 0.506^2^ |
| <2.5 | <11 | <11 |  |  | <11 | <11 | |  |  |
| 2.5–<5.0 | Redacted | Redacted |  |  | <11 | <11 | |  |  |
| 5–<10 | 41 (25.3%) | 47 (29.0%) |  |  | 15 (36.6%) | 13 (31.7%) | |  |  |
| 10–<15 | 38 (23.5%) | 36 (22.2%) |  |  | 13 (31.7%) | <11 | |  |  |
| 15–<20 | 35 (21.6%) | 36 (22.2%) |  |  | <11 | <11 | |  |  |
| 20–25 | 29 (17.9%) | 28 (17.3%) |  |  | <11 | <11 | |  |  |
| **GCA** | 23 (14.2%) | 23 (14.2%) | 0.00 | >0.999^3^ | 0 (0.0%) | 0 (0.0%) | | 0.0 | - |
| **Race, n (%)** |  |  | 0.14 | 0.435^2,3^ |  |  | | 0.27 | 0.513^2,3^ |
| White | 138 (85.2%) | 142 (87.7%) |  |  | 39 (95.1%) | 36 (87.8%) | |  |  |
| Black | <11 | <11 |  |  | <11 | <11 | |  |  |
| Other | Redacted | <11 |  |  | <11 | <11 | |  |  |
| **Region** |  |  | 0.15 | 0.863^2^ |  |  | | 0.38 | 0.408^2^ |
| West | 38 (23.5%) | 34 (21.0%) |  |  | <11 | <11 | |  |  |
| Midwest | Redacted | 16 (9.9%) |  |  | 11 (26.8%) | <11 | |  |  |
| Northeast | 55 (34.0%) | 59 (36.4%) |  |  | <11 | <11 | |  |  |
| South | 49 (30.2%) | 53 (32.7%) |  |  | <11 | 16 (39.0%) | |  |  |
| Unknown | <11 | 0 (0.0%) |  |  | 0 (0.0%) | 0 (0.0%) | |  |  |
| **Original reason for Medicare eligibility** |  |  | 0.08 | 0.480^3^ |  |  | | 0.14 | 0.532^3^ |
| Age ≥65 years | 142 (87.7%) | 146 (90.1%) |  |  | Redacted | Redacted | |  |  |
| Disabled | 20 (12.3%) | 16 (9.9%) |  |  | <11 | <11 | |  |  |
| **Charlson comorbidity index category** |  |  | 0.15 | 0.389^3^ |  |  | | 0.46 | 0.126^3^ |
| 0–1 | 70 (43.2%) | 63 (38.9%) |  |  | 19 (46.3%) | Redacted | |  |  |
| 2–3 | 59 (36.4%) | 71 (43.8%) |  |  | Redacted | 22 (53.7%) | |  |  |
| ≥4 | 33 (20.4%) | 28 (17.3%) |  |  | <11 | <11 | |  |  |
| **Time from first PMR diagnosis code to index date** |  |  | 0.07 | 0.539^1^ |  |  | | 0.02 | 0.982^1^ |
| Median (IQR) | 295.5 (142.5, 682.0) | 357.0 (129.8, 793.0) |  |  | 621.0 (268.0, 1,256.0) | 577.0 (293.0, 1,295.0) | |  |  |
| **Baseline inflammatory conditions,**  **n (%)** |  |  |  |  |  |  | |  |  |
| Asthma | 16 (9.9%) | 19 (11.7%) | 0.06 | 0.591^2,3^ | <11 | <11 | | 0.38 | 0.201^2,3^ |
| AD | <11 | <11 | 0.06 | >0.999^2^ | <11 | <11 | | 0.00 | >0.999^2^ |
| COPD | 15 (9.3%) | 18 (11.1%) | 0.06 | 0.582^2,3^ | <11 | <11 | | 0.16 | 0.712^2,3^ |
| Crohn’s disease | 0 (0.0%) | 0 (0.0%) | 0.00 | - | 0 (0.0%) | <11 | | 0.22 | >0.999^2^ |
| Psoriasis | <11 | <11 | 0.11 | >0.999^2^ | 0 (0.0%) | <11 | | 0.22 | >0.999^2^ |
| UC | 0 (0.0%) | 0 (0.0%) | 0.00 | - | 0 (0.0%) | <11 | | 0.22 | >0.999^2^ |
| Seronegative RA | 48 (29.6%) | 45 (27.8%) | 0.04 | 0.713^3^ | 29 (70.7%) | 28 (68.3%) | | 0.05 | 0.810^3^ |
| **Baseline number of Inpatient days** |  |  | 0.14 | 0.430^1^ |  |  | | 0.20 | 0.690^1^ |
| Mean (SD) | 1.3 (4.7) | 0.7 (2.4) |  |  | 0.6 (2.6) | 1.2 (3.6) | |  |  |
| Median (IQR) | 0.0  (0.0, 0.0) | 0.0  (0.0, 0.0) |  |  | 0.0  (0.0, 0.0) | 0.0 (0.0, 0.0) | |  |  |
| **Baseline number of ED visits** |  |  | 0.02 | 0.491^1^ |  |  | | 0.16 | 0.906^1^ |
| Mean (SD) | 0.5 (1.1) | 0.5 (1.0) |  |  | 0.6 (1.0) | 0.8 (1.8) | |  |  |
| Median (IQR) | 0.0  (0.0, 1.0) | 0.0  (0.0, 1.0) |  |  | 0.0  (0.0, 1.0) | 0.0 (0.0, 1.0) | |  |  |
| **Baseline number of outpatient office visits** |  |  | 0.02 | 0.765^1^ |  |  | | 0.33 | 0.059^1^ |
| Mean (SD) | 10.3 (5.9) | 10.2 (5.3) |  |  | 9.4 (5.5) | 11.0 (4.8) | |  |  |
| Median (IQR) | 9.0  (6.0, 13.0) | 9.0  (6.0, 14.0) |  |  | 8.0  (6.0, 11.0) | | 11.0  (7.0, 15.0) |  |  |
| **Calendar year of index date** |  |  | 0.14 | 0.816^2,3^ |  |  | | 0.11 | 0.991^2,3^ |
| 2016 | <11 | 12 (7.4%) |  |  | <11 | <11 | |  |  |
| 2017 | 37 (22.8%) | 34 (21.0%) |  |  | 12 (29.3%) | 14 (34.1%) | |  |  |
| 2018 | 52 (32.1%) | 52 (32.1%) |  |  | 12 (29.3%) | 11 (26.8%) | |  |  |
| 2019 | 48 (29.6%) | 48 (29.6%) |  |  | <11 | <11 | |  |  |
| 2020 | Redacted | 16 (9.9%) |  |  | <11 | <11 | |  |  |

^a^To protect patient privacy and prevent potential identification, results are reported only when the number of patients is ≥11. Additionally, data are redacted in cases where reporting results for ≥11 patients could enable derivation of counts <11. ^b^Due to small sample size, p-value was evaluated in addition to SMD to evaluate if the Cox-model required adjustment for residual cofounder; ^1^Wilcoxon rank sum test; ^2^Fisher’s exact test; ^3^Pearson’s chi-squared test.

AD, atopic dermatitis; COPD, chronic obstructive pulmonary disease; csIM, conventional synthetic immunomodulatory therapy; ED, emergency department; GC, glucocorticoid; GCA, giant cell arteritis; IL-6Ri, interleukin-6 receptor inhibitor; IQR, interquartile range; MTX, methotrexate; PMR, polymyalgia rheumatica; PS, propensity score; RA, rheumatoid arthritis; SD, standard deviation; SMD, standardized mean difference; UC, ulcerative colitis.

# Supplemental Table 6: Effectiveness primary outcomes by varying censoring rules in the IL-6Ri versus csIM and MTX subgroup

| GC discontinuation by varying censoring rules | IL-6Ri vs. csIM  N=415 | | IL-6Ri vs. MTX subgroup  N=203 | |
| --- | --- | --- | --- | --- |
|  | **HR (95% CI)** | **p-value** | **HR (95% CI)** | **p-value** |
| Enrollment end – 60 days, death, outcome, stop  index switch/add | 1.16 (0.94, 1.43) | 0.175 | 1.21 (0.90, 1.60) | 0.201 |
| Enrollment end – 60 days, 1 year, death, outcome | 1.21 (1.00, 1.45) | 0.045 | 1.08 (0.83, 1.40) | 0.573 |
| Enrollment end – 60 days, 1 year, death, outcome, switch/add | 1.25 (1.04, 1.51) | 0.020 | 1.12 (0.86, 1.47) | 0.387 |

CI, confidence interval; csIM, conventional synthetic immunomodulator; GC, glucocorticoid; HR, hazard ratio; IL-6Ri, interleukin-6 receptor inhibitor; MTX methotrexate.

# Supplemental Table 7. Reason for end of follow-up for discontinuation of GC endpoint after PS-matching

| Reason for stop follow-up, n (%) | csIM-naïve cohort (Cohort 1) | | | csIM-experienced cohort (Cohort 2) | | |
| --- | --- | --- | --- | --- | --- | --- |
|  | **IL-6Ri**  **N=187** | **csIM**  **N=187** | **p-value^a^** | **IL-6Ri**  **N=228** | **csIM**  **N=228** | **p-value^a^** |
|  |  |  | 0.068 |  |  | 0.002 |
| Event | 92 (49.2%) | 70 (37.4%) |  | 103 (45.2%) | 66 (28.9%) |  |
| Discontinue | 59 (31.6%) | 66 (35.3%) |  | 80 (35.1%) | 118 (51.8%) |  |
| 1 year end | 22 (11.8%) | 31 (16.6%) |  | 25 (11.0%) | 28 (12.3%) |  |
| Enrollment end | Redacted | <11 |  | Redacted | Redacted |  |
| Switch/add | <11 | <11 |  | <11 | <11 |  |

^a^Pearson chi square test. No patient was censored for death, as no deaths reported in the study.

To protect patient privacy and prevent potential identification, results are reported only when the number of patients is ≥11. Additionally, data are redacted in cases where reporting results for ≥11 patients could enable derivation of counts <11.

csIM, conventional synthetic immunomodulator; GC, glucocorticoid; IL-6Ri, interleukin-6 receptor inhibitor; PS, propensity score.

# Supplemental Table 8: Change in weekly prednisone-equivalent dose by duration from the index date in the IL-6Ri group, referent to csIM for MTX subgroup and sensitivity analysis (excluding GCA history) after PS-matching

| Days from Index | Difference in weekly GC dose (mg/week) (95% CI) | p-value |
| --- | --- | --- |
| MTX subgroup | | |
| Days 1–90 | -0.6 (-11.4, 10.3) | 0.917 |
| Days 91–180 | -2.7 (-15.0, 9.6) | 0.662 |
| Days 181–270 | -9.4 (-24.0, 5.2) | 0.207 |
| Days 271–365 | -13.6 (-29.7, 2.5) | 0.097 |
| Sensitivity analysis (excluding GCA history) | | |
| Days 1–90 | 0.0 (-5.4, 5.5) | 0.986 |
| Days 91–180 | -1.7 (-7.6, 4.3) | 0.585 |
| Days 181–270 | -6.7 (-13.4, -0.0) | 0.049 |
| Days 271–365 | -13.9 (-21.5, -6.3) | <0.001 |

*Patients with available data with ≥1 day in the interval. Patients were censored for death, enrollment end – 60 days, index date + last day of assessment period, stopping index therapy, switching/adding therapy. A difference of 1 unit in in the above table represents 1 mg change in weekly PED dose between the exposure group (IL-6Ri) and the comparator (csIM). Negative numbers reflect a greater reduction in weekly PED dose in the IL-6Ri-treated patients versus the csIM and MTX group.

CI, confidence interval; csIM, conventional synthetic immunomodulator; GC, glucocorticoid; GCA, giant cell arteritis; IL-6Ri, interleukin-6 receptor inhibitor; MTX, methotrexate; PED, prednisone-equivalent dose; PS, propensity score.

# Supplemental Table 9. Outcomes at year 1 for PS-matched patients included in the sensitivity analysis (excluding patients with history of GCA) and receiving IL-6Ri or csIM therapies in the csIM-naïve or csIM-experienced cohort

| Outcomes at 1 year | csIM-naïve cohort  (no GCA history before direct-matching) | | | csIM-experienced cohort  (no GCA history before direct-matching) | | |
| --- | --- | --- | --- | --- | --- | --- |
|  | **IL-6Ri**  **N=183** | **csIM**  **N=183** | **p-value^a^** | **IL-6Ri**  **N=268** | **csIM**  **N=268** | **p-value^a^** |
| Patients with GC discontinuation,  n (%) | 91 (49.7%) | 64 (35.0%) | 0.004 | 122 (45.5%) | 76 (28.4%) | <0.001 |
| Patients with minimal GC use, n (%) | 101 (55.2%) | 68 (37.2%) | <0.001 | 127 (47.4%) | 86 (32.1%) | <0.001 |
| Average weekly GC dose (mg per person-weeks) |  |  | 0.004 |  |  | 0.094 |
| Mean (SD) | 45.7 (34.4) | 53.7 (32.9) |  | 46.4 (37.4) | 49.7 (32.7) |  |
| Median (IQR) | 36.0  (20.9, 63.2) | 47.7  (28.0, 74.8) |  | 38.9  (24.5, 63.0) | 41.5  (29.5, 66.1) |  |

Minimal GC defined as prednisone-equivalent dose of ≤2 mg/day or stop GC; GC dose expressed as prednisone equivalents.

^a^Pearson chi square test for categorical variables, and Wilcoxon rank sum test for continuous variables.

csIM, conventional synthetic immunomodulator; GC, glucocorticoid; GCA, giant cell arteritis; IL-6Ri, interleukin-6 receptor inhibitor; IQR, interquartile range; PS, propensity score; SD, standard deviation.

# SUPPLEMENTAL FIGURE 1: HR^a^ (95% CI) for patients on IL-6Ri versus csIM therapy included in the sensitivity analysis (excluding patients with history of GCA before direct-matching) after PS-matching

Minimal GC dose defined as prednisone-equivalent dose of ≤2 mg/day or stop GC.

^a^Adjusted for sex, baseline and index day prednisone-equivalent dose category, asthma, atopic dermatitis, COPD, number of emergency department visits, original reason for Medicare eligibility, and Charlson comorbidity index category.

CI, confidence interval; COPD, chronic obstructive pulmonary disease; csIM, conventional synthetic immunomodulator; GC, glucocorticoid; GCA, giant cell arteritis; HR, hazard ratio; IL-6Ri, interleukin-6 receptor inhibitor; PS, propensity score.
